# Green synthesis of Ag/MgO nanocomposites from red pepper seed waste for efficient sunlight-driven diclofenac degradation: experimental investigation and EEFO-optimized DT_LSBoost predictive modeling

**DOI:** 10.1039/d6ra06722k

**Published:** 2026-09-14

**Authors:** Sabrina Mechati, Meriem Zamouche, Hichem Tahraoui, Nasreddine Hadjadj, Mohammed Kebir, Sonia Cherif, Jie Zhang, Lotfi Mouni, Abdeltif Amrane

**Affiliations:** a Laboratoire de Recherche sur le Médicament et le Développement Durable (ReMeDD), Department of Environmental Engineering, Salah Boubnider University of Constantine 3 Constantine Algeria meriem.zamouche@univ-constantine3.dz; b Laboratoire de Génie de l'environnement et des Procédés Pharmaceutique LGEPP, University of Setif 1 Algeria; c Laboratory of Biomaterials and Transport Phenomena (LBMTP), Yahia Fares University Medea 26000 Algeria; d Centre de Recherche en Biotechnologie Ali Mendjli Nouvelle Ville UV 03 BP E73 Constantine Algeria; e Research Unit in Physical and Chemical Analysis in the Fluid and Solid Medium (URAPC-MFS/CRAPC) Bou-Ismail 42000 BP 384 Algeria; f Laboratory of Reaction Engineering, Faculty of Mechanical Engineering and Process Engineering, USTHB BP 32 Al Alia Algiers 16111 Algeria; g School of Engineering, Merz Court, Newcastle University Newcastle Upon Tyne NE1 7RU UK; h Laboratory for Natural Resources Management and Quality Assurance, Akli Mohand University Oulhadj Bouira Algeria; i Univ Rennes, Ecole Nationale Supérieure de Chimie de Rennes, CNRS, ISCR (Institut des 17 Sciences Chimiques de Rennes) – UMR 6226 F-35000 Rennes France

## Abstract

The occurrence of pharmaceutical residues in aquatic environments has become a major environmental concern due to their persistence, low biodegradability, and incomplete removal by conventional wastewater treatment processes. Among these contaminants, diclofenac (DCF) is frequently detected in surface and municipal wastewaters, highlighting the need for efficient and sustainable remediation technologies. In this study, Ag/MgO NCs were green synthesized using *Capsicum annuum* L. (red pepper) seed extract as a natural reducing and capping agent. Physicochemical characterization by XRD, FTIR, SEM-EDX, UV-Vis DRS, and pH_PZC_ analyses confirmed the successful formation of the Ag/MgO NCs, with crystallite sizes ranging from 12.0 to 9.0 nm and an extended visible-light absorption edge shifting to an apparent threshold of 1.83 eV due to localized surface plasmon resonance (LSPR) and interfacial states, thereby significantly enhancing solar-light utilization. The photocatalytic performance under natural sunlight was systematically evaluated by investigating the effects of irradiation time, catalyst dosage, Ag loading, solution pH, initial DCF concentration, electrolyte type and concentration, and irradiation source. The 10% Ag/MgO photocatalyst exhibited the highest activity, achieving 80% degradation of 10 mg L^−1^ DCF within 180 min under optimum operating conditions. Radical trapping experiments identified hydroxyl radicals (˙OH) as the dominant reactive species, while metallic Ag acted as an efficient electron sink, suppressing charge-carrier recombination and enhancing photocatalytic efficiency. To accurately predict the degradation performance, a Decision Tree coupled with Least-Squares Boosting (DT-LSBoost) model was developed and its hyperparameters were optimized using the Electric Eel Foraging Optimization (EEFO) algorithm. The optimized model demonstrated excellent predictive capability (*R* = 0.9999; RMSE = 1.2 × 10^−3^), while residual analysis confirmed its robustness and generalization ability. A MATLAB-based graphical user interface was further developed to enable rapid prediction of photocatalytic performance under different operating conditions. The integration of green-synthesized Ag/MgO NCs with an EEFO-optimized DT-LSBoost model provides a novel and efficient framework for predicting solar-driven diclofenac degradation. This integrated strategy highlights the potential of combining green nanotechnology with explainable machine learning to support the design, optimization, and practical implementation of sustainable photocatalytic water-treatment systems.

## Abstract 2

Biogenic Ag/MgO nanocomposites synthesized using pepper extract show enhanced photocatalytic degradation of diclofenac under sunlight irradiation.

## Graphical abstract

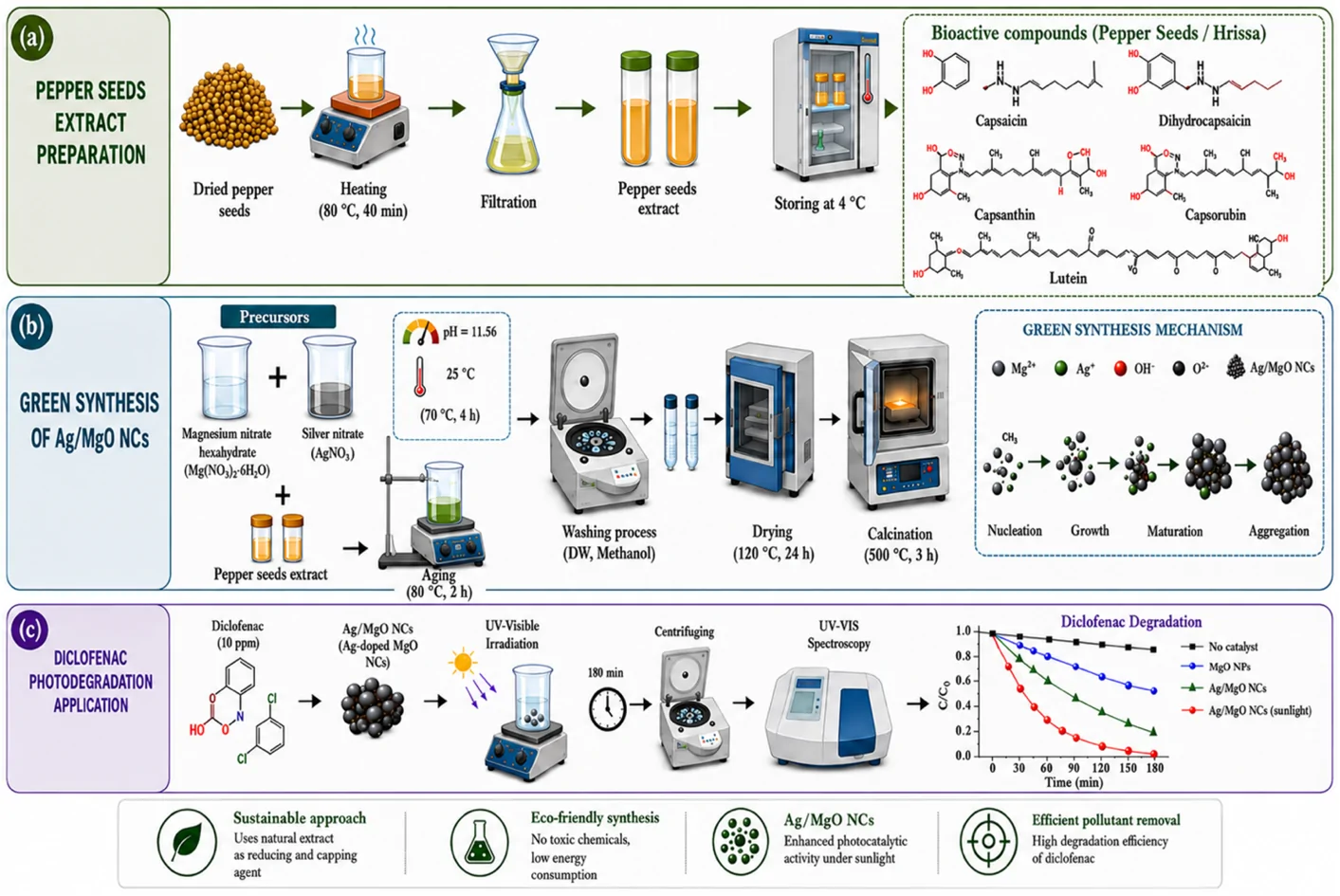

## 1. Introduction

The presence of persistent traces of pharmaceutical residues has become a major environmental concern over the last decades,^1^ particularly non-steroidal anti-inflammatory drugs (NSAIDs), have been extensively reported in surface waters, sediments, and soils across rivers and agricultural areas, highlighting their widespread distribution and long-term environmental persistence.^2,3^ Among them, diclofenac (DCF) is one of the most frequently detected NSAIDs in aquatic systems due to its extensive consumption,^4^ continuous discharge, and resistance to conventional wastewater treatment processes.^5^ DCF is characterized by low biodegradability,^2^ high chemical stability, and pronounced ecotoxicological effects,^6,7^ even at trace concentrations, which has led to its classification as a hazardous emerging contaminant. Its presence has been associated with chronic toxicity to aquatic organisms,^8^ endocrine disruption, and bioaccumulation,^9^ raising significant concerns regarding environmental safety and public health.

Existing conventional wastewater treatment technologies for DCF removal, including biological treatment, adsorption,^10,11^ membrane separation,^1^ Fenton oxidation, and electrochemical processes, often face limitations such as slow kinetics, high costs, fouling, generation of secondary pollutants, or incomplete degradation. These approaches generally struggle with non-biodegradable pharmaceuticals, leading to partial removal and potential secondary contamination, contributing to environmental risks like pathogen resistance and endocrine disruption.^1^

To overcome these challenges, advanced oxidation processes (AOPs) have emerged as efficient alternatives. Among them, heterogeneous photocatalysis has gained significant attention due to its high degradation efficiency, operational simplicity, and compatibility with renewable energy.^12,13^ Photocatalysis generates reactive oxygen species (ROS), such as hydroxyl (˙OH) and superoxide (˙O_2_^−^) radicals,^14^ which oxidize complex pharmaceutical molecules and mineralize them into harmless products like carbon dioxide and water, without forming harmful by-products.^15^

Furthermore, sunlight-driven photocatalysis offers additional advantages by eliminating the need for artificial irradiation, reducing energy consumption, and lowering operational costs.^16^ Compared with UV-based systems, visible-light photocatalysis is more energy-efficient, requires no specialized equipment, and is suitable for large-scale applications. It also minimizes toxic intermediates and achieves faster degradation than biological treatments, highlighting its potential as a sustainable solution for pharmaceutical-contaminated wastewater.^17^

Magnesium oxide (MgO) nanoparticles (NPs) have emerged as promising photocatalysts due to their low toxicity, chemical stability, and affordability. However, pristine MgO suffers from a wide band gap and rapid electron–hole recombination, which limit its photocatalytic efficiency, particularly under visible light. To address these limitations, researchers have explored doping MgO with transition metal ions to modify its electronic structure and enhance its photocatalytic performance.^18,19^

Among various dopants, silver (Ag) is particularly effective. Although costly, Ag not only reduces the band gap and improves light absorption through surface plasmon resonance but also suppresses electron–hole recombination. Consequently, Ag/MgO NCs exhibits enhanced photocatalytic activity along with additional antibacterial properties, highlighting its multifunctional potential. Evaluating whether very small concentrations of Ag can significantly improve MgO's photocatalytic efficiency is of particular interest, as this could provide an economically viable approach for sunlight-driven remediation of pharmaceutical pollutants.^20^

Despite extensive studies on dye degradation under artificial UV light, systematic investigations of Ag-doped MgO for the removal of pharmaceutical contaminants such as diclofenac under natural sunlight remain limited. Moreover, the influence of operational parameters relevant to real water matrices.

In addition, photocatalytic degradation processes involve complex and highly non-linear interactions between material properties and operational conditions, making accurate prediction and optimization challenging using conventional kinetic approaches.^21^ Recently, artificial intelligence (AI) techniques, have emerged as powerful tools for modeling advanced oxidation processes. AI are capable of capturing complex non-linear relationships between multiple input variables and degradation efficiency without requiring explicit mechanistic assumptions, offering high predictive accuracy and robustness.^22^ Although interest in AI-assisted photocatalysis is growing, comprehensive studies combining experiments and Decision Tree coupled with Least-Squares Boosting (DT_LSBoost) modeling of diclofenac degradation under sunlight with Ag/MgO NCs nanoparticles are lacking. Integrating experimental optimization with DT_LSBoost modeling can enhance understanding, identify key parameters, and improve photocatalytic treatment scalability.

In this work, Ag/MgO nanocomposites were successfully synthesized through an environmentally friendly route using *Capsicum annuum* L. (red pepper) seed extract as a natural reducing and stabilizing agent. The synthesized nanomaterials were comprehensively characterized to elucidate their structural, morphological, elemental, optical, and surface properties. Their photocatalytic performance toward diclofenac degradation was systematically investigated under natural sunlight by evaluating the influence of key operating parameters, including solution pH, catalyst dosage, initial pollutant concentration, electrolyte type and concentration, and irradiation conditions. In addition, the photocatalytic mechanism was explored through radical scavenging experiments to identify the predominant reactive species involved in the degradation process and Total Organic Carbon (TOC) analysis to identify the predominant reactive species and quantify the true mineralization yield. Furthermore, a Decision Tree coupled with Least-Squares Boosting (DT_LSBoost) machine learning model was developed to predict the photocatalytic degradation efficiency based on the experimental operating conditions. To maximize its predictive capability, the model hyperparameters were automatically optimized using the Electric Eel Foraging Optimization (EEFO) algorithm, and a MATLAB-based graphical user interface (GUI) was implemented to facilitate practical application of the developed predictive framework.

The novelty of this work lies in the integration of green nanomaterial synthesis, solar photocatalysis, and artificial intelligence into a unified framework for pharmaceutical wastewater treatment. Unlike previous studies that primarily focus on either photocatalyst development or predictive modeling, this study combines the green synthesis of Ag/MgO nanocomposite from agricultural waste, a comprehensive evaluation of diclofenac degradation under natural sunlight, and the development of an EEFO-optimized DT_LSBoost predictive model. Furthermore, the implementation of a MATLAB-based decision-support application extends the practical applicability of the proposed approach, providing a rapid and reliable tool for predicting photocatalytic performance under various operating conditions while supporting the design and optimization of sustainable water treatment processes.

## 2. Experimental

### 2.1. Reagents

Pepper seeds were primarily collected as a byproduct during the extraction and sieving stage of harissa production at the CAB (Amor Benamor) cannery located in Guelma, in northeastern Algeria (36°30′20″ N, 7°25′32″ E). Magnesium nitrate hexahydrate (Mg (NO_3_) _2_·6H_2_O, 99.99%), sodium hydroxide (NaOH, 97%), and silver nitrate (AgNO_3_, ≥99.9%) were used as precursor chemicals for the synthesis of MgO-NPs and Ag/MgO NCs. All chemicals were purchased from Sigma-Aldrich and used as received without further purification. Distilled water (DW) was employed throughout all experiments. For the photocatalytic and scavenger studies, *tert*-butanol (T-BuOH), benzoquinone (BQ), ammonium oxalate (AO), and silver nitrate (AgNO_3_) were used as radical scavengers to identify the main reactive species involved in the photodegradation process.

### 2.2. Preparation of aqueous pepper seed extract

After being thoroughly washed with running water to remove external impurities and industrial additives and subsequently rinsed with distilled water, the pepper seeds were air-dried for 2 days. The dried material was then mechanically ground using a laboratory mill and sieved to obtain a homogeneous particle size of approximately 200 µm.

**Fig. 1:**
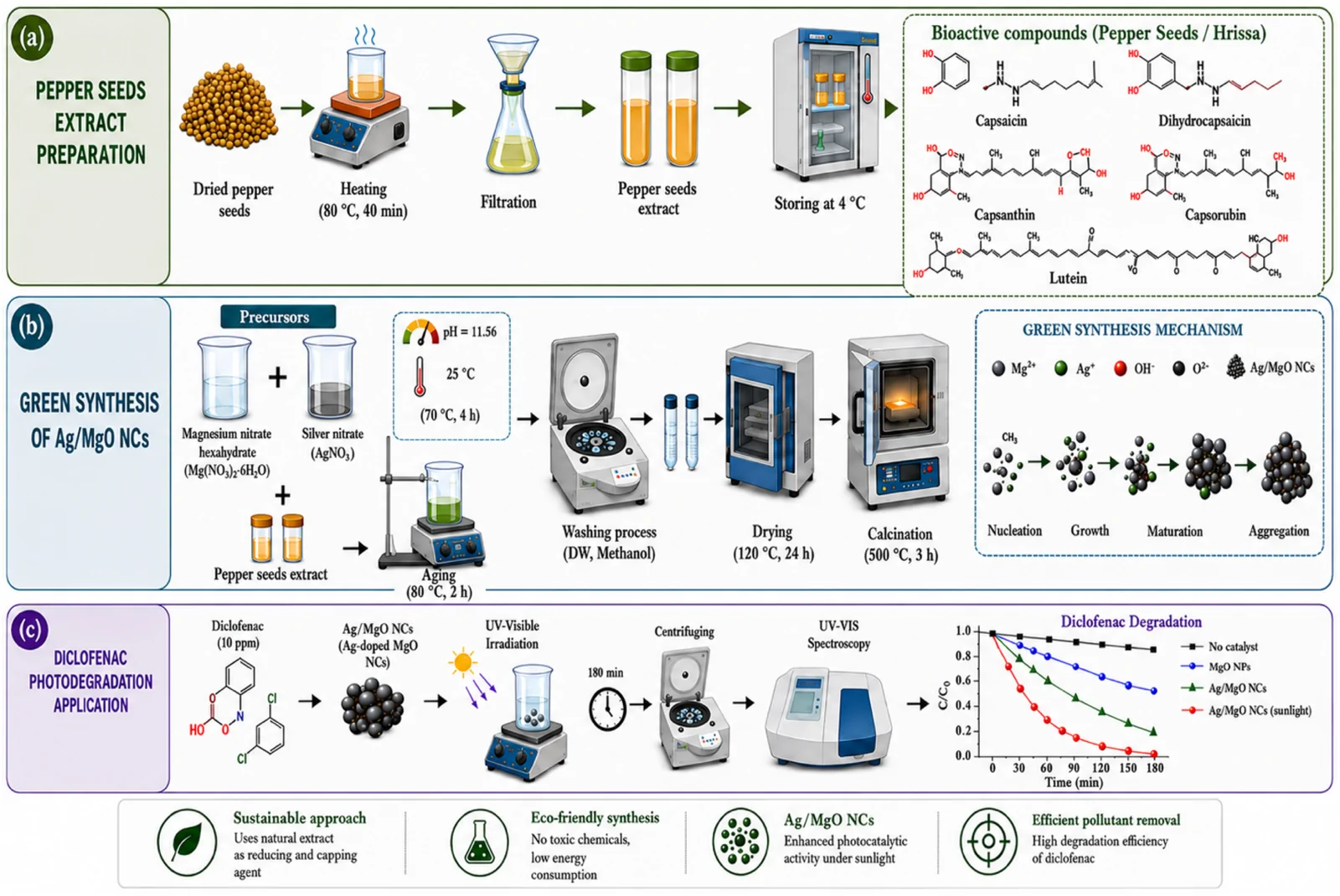
Schematic representation of (a) pepper seed extract preparation process (b) biogenic synthesis process of NPs (c) sunlight degradation of DCF.

Subsequently, 10 g of the dried milled pepper seeds were dispersed in 100 mL of distilled water and heated at 80 °C under continuous stirring (350 rpm) for 40 min. The resulting mixture was filtered using Whatman No. 1 filter paper, and the obtained extract was stored at 4 °C for further use. As illustrated in Fig. 1(a), this process yielded a clear extract, which was employed for the green synthesis of nanoparticles.

### 2.3. Biogenic synthesis of MgO-NPs and Ag-doped MgO NPs

MgO-NPs and Ag/MgO NCs were synthesized *via* a green route by combining metal precursor solutions with pepper seed extract (Fig. 1(b)). In a typical synthesis, the required amounts of magnesium nitrate hexahydrate (Mg (NO_3_)_2_·6H_2_O) and, for the doped samples, silver nitrate (AgNO_3_) were first dissolved in distilled water to constitute 80% of the total reaction volume. The remaining 20% consisted of the pepper seeds aqueous extract, which was added dropwise to the precursor solution under continuous stirring (350 rpm). The resulting mixture was heated at 80 °C for 2 h, during which the gradual formation of a brown to dark brown precipitate was observed, indicating the formation of MgO-NPs and Ag/MgO NCs, respectively.

The reaction suspensions were aged at 70 °C for 4 h, then ultrasonicated for 20 min at 40 °C to improve dispersion. After cooling, products were collected by centrifugation, washed with distilled water and methanol, dried at 120 °C under vacuum for 24 h, and calcined at 500 °C for 3 h to enhance crystallinity and remove residual organics.

### 2.4. Characterization of the catalysts

The successful synthesis of pure MgO-NPs and Ag/MgO NCs using pepper seeds extract was confirmed through comprehensive physicochemical characterization of their structural, morphological, elemental, and optical properties.

The crystalline structure and phase purity were analyzed using a Malvern Panalytical Empyrean diffractometer equipped with monochromatic Cu Kα radiation (*λ* = 1.5406 Å). The analysis was conducted at an operating voltage of 45 kV and a current of 40 mA, scanning the *θ* range from 5° to 80°. The average crystallite size (*D*) was calculated using the Debye–Scherrer equation (eqn (1)):^23^1
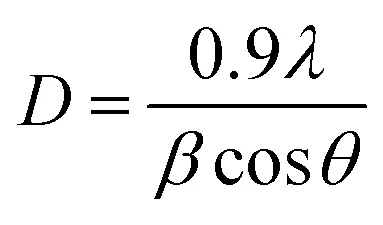
where *λ* is the X-ray wavelength (0.1541 nm), *β* is the full width at half maximum (FWHM) of the diffraction peak, and *θ* is the Bragg diffraction angle.

The morphology, particle shape, and elemental composition of the synthesized pure MgO-NPs and Ag/MgO NCs were analyzed using scanning electron microscopy coupled with energy-dispersive X-ray spectroscopy (SEM–EDS, FEI Quanta 250 FEG, FEI Company, the Netherlands). The microscope was equipped with a Schottky field emission gun (FEG) and operated at an accelerating voltage of 10 kV. The instrument provides a spatial resolution of 1.2 nm under high-vacuum conditions. SEM images were acquired at magnifications ranging from 5000× to 120 000×.

The identification of functional groups in the prepared oxides was performed by Fourier-transform infrared (FTIR) spectroscopy (Shimadzu, Japan). The spectra were recorded over a wavenumber range of 400–4000 cm^−1^.

UV-Vis diffuse reflectance spectra (DRS) were recorded using a Shimadzu UV-2401 PC spectrophotometer, with barium sulfate as the reference material. Measurements were performed at room temperature over a wavelength range of 200–600 nm. The band gap energies were determined using the Kubelka–Munk function eqn (2):^24^2
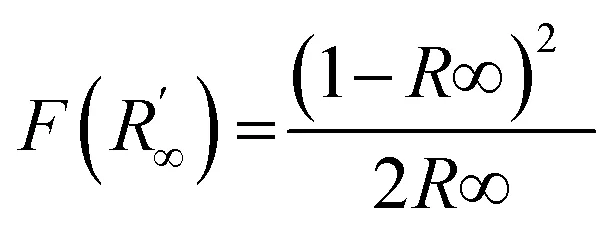
where *R*_∞_ represents the absolute reflectance value, and 
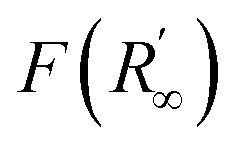
 corresponds to the absorption coefficient. The direct band gap of the nanoparticles was calculated by plotting the transformed function 
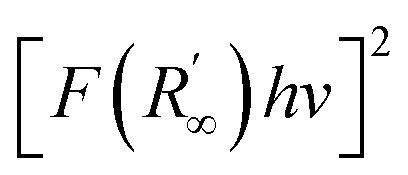
*versus* hν (eV). The linear region of the Tauc plot was extrapolated to the intercept where 
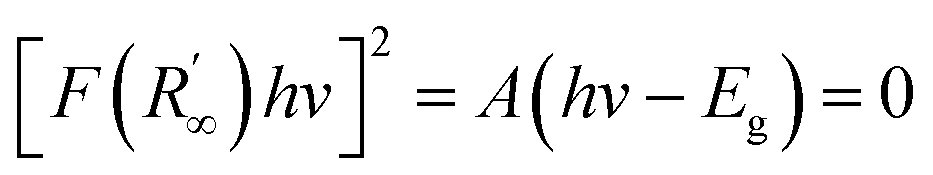
, corresponding to the direct band gap energy *E*_g_

The pH of the point of zero charge (pH_P*Z*C_) of the samples was determined using the pH-drift method.^25,26^ A 0.01 M NaCl solution was prepared, and its initial pH values were adjusted between 2 and 12 using NaOH (0.1 M). A fixed catalyst dosage of 0.5 g L^−1^ was used.

The suspensions were stirred and allowed to equilibrate for 48 hours at room temperature. The pH_P*Z*C_ corresponds to the pH at which the final pH equals the initial pH, indicating a neutral surface with no net charge. This parameter is essential for understanding the interaction between catalysts and ionic species.^27^

### 2.5. Photocatalytic activity evaluation

The photocatalytic performance of pure MgO-NPs and Ag/MgO NCs was evaluated in a borosilicate beaker under solar light irradiation (Fig. 1c). The reaction system was open to atmospheric air; therefore, dissolved oxygen from the ambient environment acted as the electron acceptor during the photocatalytic process. A catalyst dosage of 0.5 g L^−1^ was used in 10 ppm diclofenac (DCF) solution.

Prior to irradiation, the photocatalyst suspension was magnetically stirred in the dark for 30 min under ambient conditions to establish adsorption–desorption equilibrium between the catalyst surface and DCF molecules. Following this equilibration period, the photocatalytic reaction was conducted under solar irradiation for a total duration of 180 min. Throughout the irradiation period, the reaction temperature was maintained at approximately 27 °C by means of an external cold-water circulation system, thereby minimizing thermal effects on the photocatalytic process.

The experiments were carried out under clear-sky summer conditions (June–July, 11:00 AM–2:00 PM) in Constantine, Algeria (36°14′N, 6°37′E), corresponding to a UV index of 8–9 and an average clear-sky solar irradiance of 850 ± 50 W m^−2^. Meteorological data and incident solar radiation parameters were obtained from local weather station records, complemented by satellite-derived solar irradiance data from the PVGIS database (European Commission), to ensure accurate characterization of the solar exposure conditions.

To investigate the contribution of the reactive species generated under irradiation to DCF degradation, trapping experiments were performed by adding selected scavengers to the reaction mixture, namely *tert*-butanol (TBA) (˙OH scavenger), AgNO_3_ (e^−^ scavenger), benzoquinone (O_2_˙^−^ scavenger), and Na_2_C_2_O_4_ (h^+^ scavenger).

At regular 30 min intervals throughout the 180 min reaction, 1 mL aliquots were withdrawn and transferred into 1.5 mL microcentrifuge tubes (Eppendorf). The samples were then subjected to centrifugation at 4000 rpm for 8 min to separate the catalyst. The resulting supernatant was carefully collected and analyzed by UV-visible spectroscopy over the 200–600 nm wavelength range. The DCF degradation yield (%) was calculated using eqn (3):^28^3
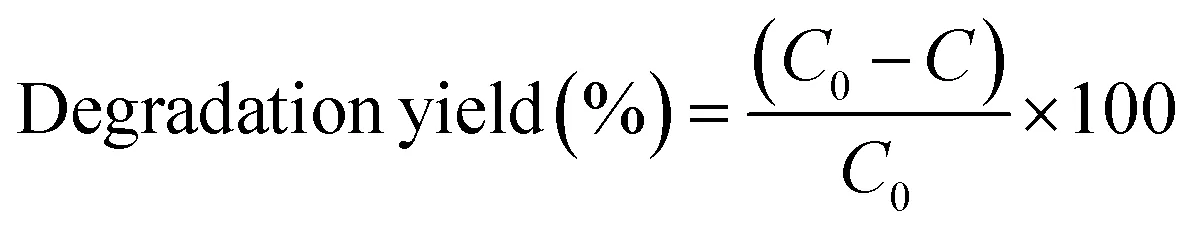
where *C*_0_ and *C* represent the initial and residual DCF concentrations at time *t*.

### 2.6. Reusability

The reusability and operational stability of the 10 wt% Ag/MgO NCs were evaluated over five consecutive photocatalytic cycles under identical experimental conditions. After each cycle, the photocatalyst was separated from the reaction mixture by centrifugation (4000 rpm, 10 min). To eliminate residual adsorbed diclofenac molecules and degradation intermediates, the recovered solid was thoroughly washed three times with alternating cycles of distilled water and methanol, followed by a final rinsing with distilled water. The collected nanoparticles were subsequently dried in an oven at 120 °C for 4 h prior to the next photocatalytic run. The residual photocatalytic degradation efficiency was monitored after each cycle to evaluate potential activity loss and determine the practical feasibility of the catalyst for sustainable wastewater treatment.

### 2.7. Machine learning framework based on the DT_LSBoost model

Artificial intelligence has become an efficient alternative to conventional mathematical modeling for describing complex environmental processes involving multiple interacting variables. Unlike empirical or mechanistic equations, machine-learning algorithms establish predictive relationships directly from experimental observations without requiring prior assumptions regarding the physical mechanisms governing the studied system. Among the available supervised learning methods, decision tree (DT) regression has attracted considerable attention because it combines computational simplicity, high interpretability, and the capability to reproduce nonlinear behaviors encountered in chemical and environmental engineering applications.^29,30^ The operating principle of a decision tree is based on successive partitioning of the predictor space. Beginning with the complete dataset, the algorithm repeatedly identifies the most informative splitting criterion to divide the observations into increasingly homogeneous groups. This recursive process continues until predefined stopping conditions are satisfied, producing a hierarchical model in which every terminal node represents a local prediction. Because each prediction originates from explicit decision rules, DT models allow direct interpretation of the influence of each experimental variable on the system response. Nevertheless, a single regression tree generally suffers from limited predictive robustness. Excessive tree growth often produces models that accurately reproduce calibration data but fail to maintain satisfactory accuracy when confronted with new observations. Moreover, slight perturbations in the training dataset may substantially modify the generated tree topology, resulting in unstable predictions.^29,30^ To improve prediction reliability, the present work employed the Least-Squares Boosting (LSBoost) strategy as an ensemble learning framework. Contrary to conventional regression trees, LSBoost does not rely on a single predictive structure. Instead, it sequentially generates a collection of relatively simple regression trees that cooperate to estimate the desired response. During every boosting cycle, the residual errors obtained from the current ensemble are calculated, and a new learner is specifically trained to reduce these remaining inaccuracies. The contribution of each learner is subsequently incorporated into the global predictor, allowing the overall model to progressively converge toward the experimental observations. By iteratively minimizing the prediction residuals, the LSBoost algorithm considerably enhances predictive precision, decreases model variance, and improves extrapolation capability while maintaining resistance to overfitting.^29,30^

This hybrid DT_LSBoost framework was adopted to predict the evolution of diclofenac during photocatalytic degradation using Ag/MgO nanocomposite synthesized through a green route. The target variable corresponded to the normalized residual concentration ratio (*C*/*C*_0_), while the predictor matrix consisted of eight experimental descriptors characterizing the operating conditions. These descriptors included irradiation time, initial pH, catalyst loading, silver content incorporated into the MgO nanoparticles, initial diclofenac concentration, electrolyte identity, electrolyte concentration, and illumination condition. Together, these variables describe the principal physicochemical factors controlling photocatalytic degradation efficiency. Since machine learning algorithms require numerical representations of categorical information, the qualitative experimental variables were transformed before model development. The electrolyte composition was encoded using integer values, where 0 indicated experiments performed without dissolved salts, 1 represented sodium chloride (NaCl), and 2 corresponded to potassium chloride (KCl). Likewise, illumination conditions were converted into numerical categories by assigning the values 0, 1, 2, and 3 to natural sunlight, ultraviolet irradiation (26 W), halogen irradiation (150 W), and dark experiments, respectively. In contrast, electrolyte concentration remained a continuous numerical variable expressed in parts per million, covering the concentration interval investigated experimentally.

Prior to training, all quantitative variables were subjected to feature scaling through linear normalization within the interval of −1 to +1. This preprocessing step minimizes the influence of differences in measurement units among predictors, improves numerical stability, and accelerates the convergence of the learning procedure. Following normalization, the complete dataset was randomly divided into three mutually exclusive subsets. Seventy percent of the available observations were allocated to parameter learning, whereas the remaining data were equally reserved for testing and independent validation. This strategy allows the predictive capability of the developed model to be assessed using observations that were not involved during model calibration.

Model performance strongly depends on the appropriate selection of hyperparameters controlling both the regression trees and the boosting procedure. Consequently, automatic hyperparameter optimization was performed using the Electric Eel Foraging Optimization (EEFO) algorithm. EEFO is a population-based metaheuristic inspired by the cooperative hunting behavior and adaptive navigation mechanisms of electric eels.^31^ Owing to its effective balance between exploration of unexplored regions and exploitation of promising candidate solutions, EEFO is particularly suitable for solving nonlinear optimization problems involving multiple interacting parameters. During the optimization stage, the algorithm simultaneously identified the optimal minimum leaf size, minimum parent size, maximum number of tree splits, learning rate, and boosting cycles. Automatic adjustment of these parameters allowed the DT_LSBoost framework to achieve an optimal compromise between prediction accuracy, computational efficiency, and model generalization.

The final predictive model was quantitatively assessed using two complementary statistical performance indicators, namely the correlation coefficient (*R*) and the root mean square error (RMSE).^32–35^ The correlation coefficient measures the consistency between calculated and experimental responses, whereas RMSE evaluates the average prediction error over the entire dataset. High *R* values associated with low RMSE values therefore indicate that the optimized DT_LSBoost model successfully reproduces the experimental behavior of diclofenac photocatalytic degradation over the investigated operating domain ^36–40^

## 3. Results and discussions

### 3.1. Mechanism of synthesis

**Fig. 2:**
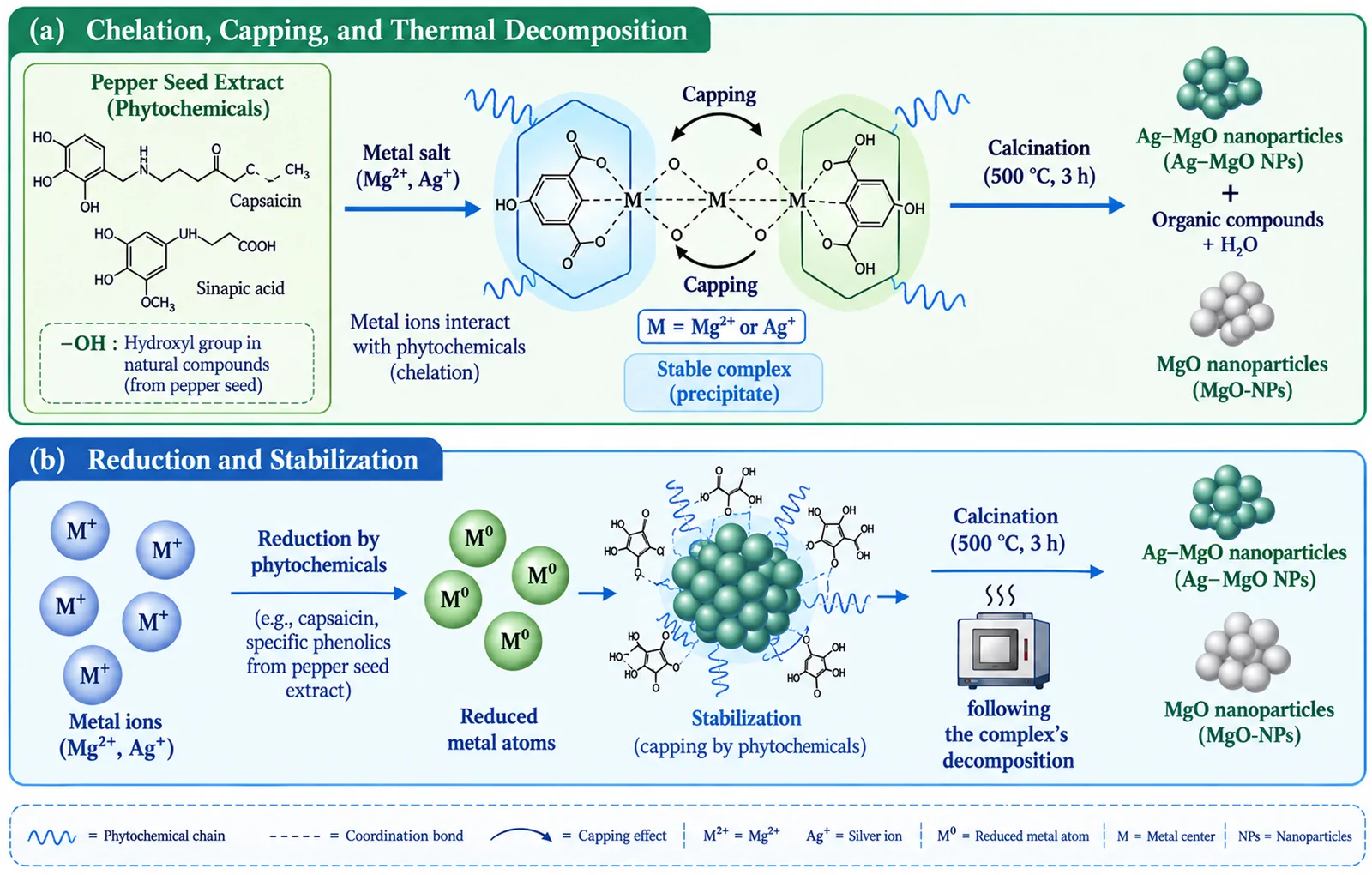
Proposed mechanisms for the green synthesis of MgO and Ag–MgO nanoparticles using pepper seed extract: (a) chelation, capping, and thermal decomposition; (b) reduction and stabilization.

As shown in Fig. 2 the green synthesis of MgO-Nps and Ag/MgO NCs using pepper seed extract follows a dual mechanism of chelation and reduction. Phytochemical screening reveals bioactive compounds such as polyphenols, flavonoids, capsaicinoids, and essential fatty acids,^41^ which contain functional groups (–OH, –NH_2_, –COOH, C=O) that act as reducing and capping agents.^42^

The nanocomposite forms *via* two synergistic routes:

(a) Chelation and stabilization: oxygen-rich functional groups chelate Mg^+2^ ions, forming a stable organometallic complex that prevents particle agglomeration. Calcination decomposes this framework, producing pure, crystalline MgO nanoparticles.^43^

(b) Bioreduction: phenolic compounds reduce Ag^+^ to metallic Ag or stabilize Ag^+^ in the MgO lattice, while the extract coats nanoparticles, controlling crystal growth and ensuring uniform Ag dispersion in the MgO matrix.^43^

### 3.2. Characterization

#### 3.2.1. X-ray diffraction analysis (XRD)

The crystalline phase, purity, and structural integrity of synthesized nanoparticles were analyzed using X-ray diffraction (XRD) to confirm the MgO lattice formation and the presence of silver dopants. This analysis allows for determination of the average crystallite size and lattice parameters, offering insights into structural modifications from the green synthesis method and silver incorporation.

**Fig. 3:**
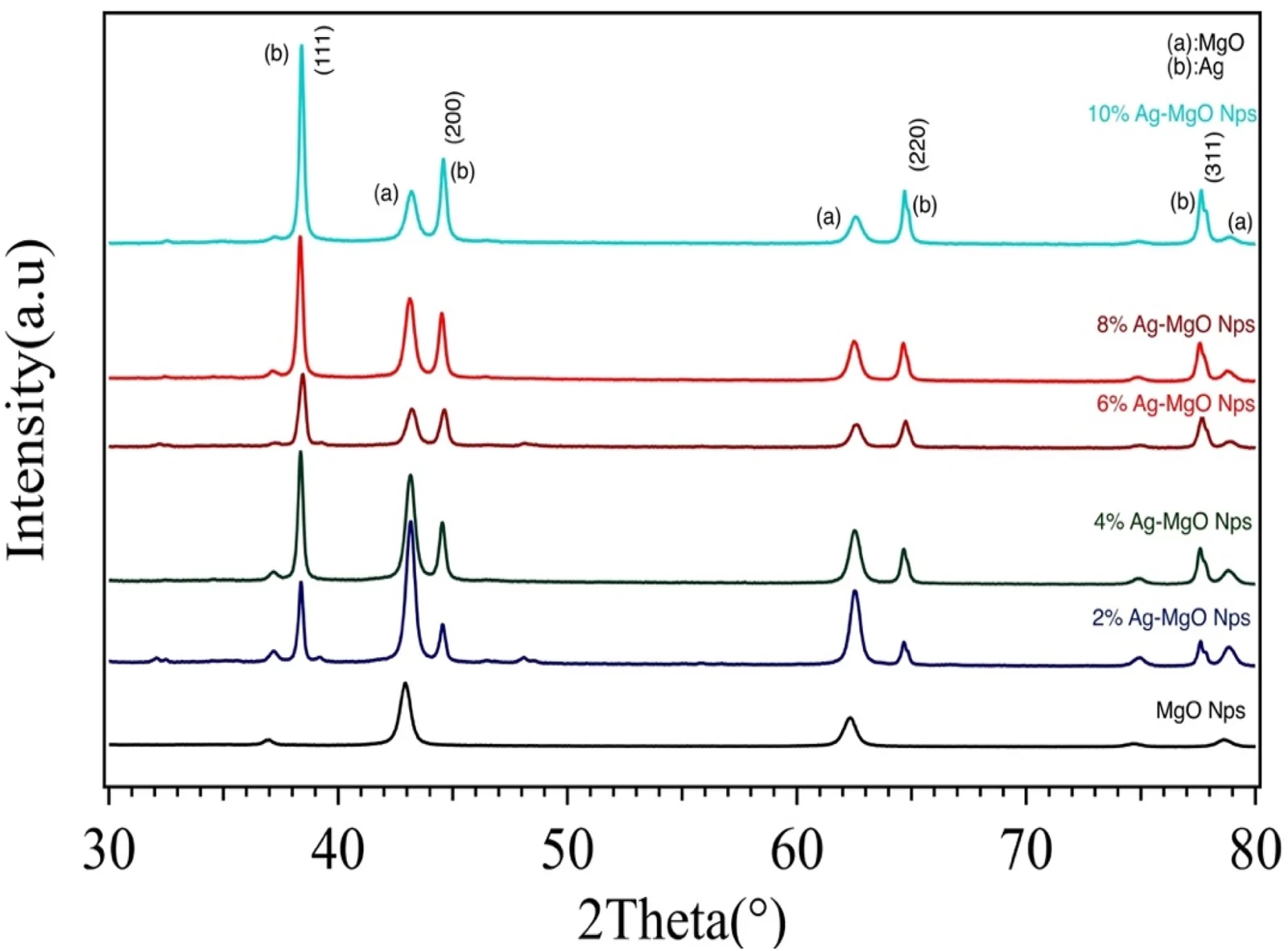
XRD spectra of pure MgO-NPs and (2–10%) Ag/MgO NCs: (a) MgO and (b) Ag.

**Fig. 4:**
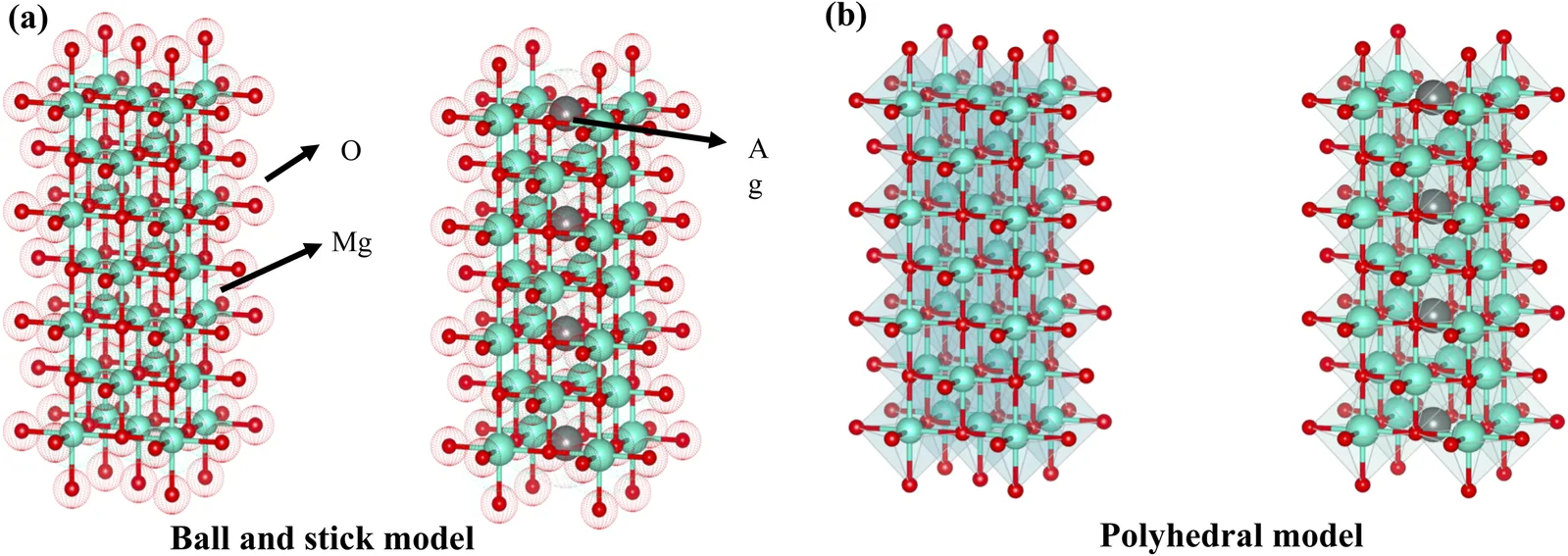
Visual representation of the face-centered cubic (FCC) periclase structure of pure MgO-NPs and Ag/MgO NCs: (a) Ball-and-stick model, (b) polyhedral model.

The phase purity and crystalline structure of pure and (2–10%) Ag/MgO NCs were analyzed by X-ray diffraction (XRD), as shown in Fig. 3. Pristine MgO displayed distinct peaks at 2*θ* values of 42.69°, 62.3°, and 78.6°, corresponding to the (200), (220), and (311) planes, matching JCPDS card No. 45-0946 and confirming an FCC periclase MgO structure. Fig. 4(a and b) Illustrates the cubic periclase crystallographic structure of the MgO-NPs and Ag/MgO NCs, confirming the absence of hexagonal phases, new diffraction peaks at 2*θ* ≈ 38.1° and 44.3° (assigned to Ag (111) and (200) planes) appear and intensify as Ag content increases from 2% to 10%, confirming successful Ag/MgO NCs formation. These extra peaks indicate the presence of secondary phase clusters caused by the accumulation of face-centered cubic silver (Ag).^44^ It is important to mention that the crystalline phases of silver and MgO emerge generally beyond 300 °C.^45^

As Ag concentration increases, the MgO (200) diffraction peak shifts to higher 2*θ* values, moving from 42.69° for pure MgO to 42.91° for the 10% Ag-doped sample. This shift is accompanied by a decrease in the lattice constant (*α*) from 4.212 Å to 4.194 Å and a reduction in cell volume (*V*), indicating lattice distortion and microstrain. The lattice parameters were calculated using the following relation (eqn (4)–(6)):^46^4
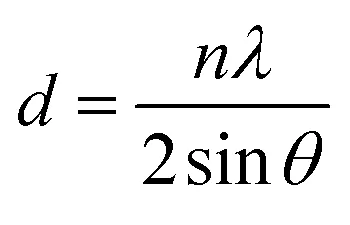
5
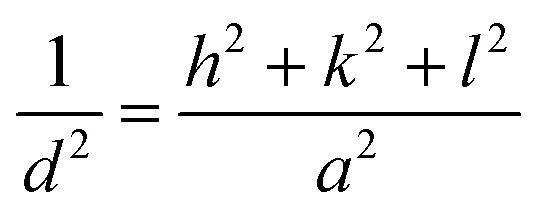

6 *V* = *a*^3^

where *d* is the interplanar spacing, *λ* is the X-ray wavelength (1.5406 Å), (*hkl*) are the Miller indices, *a* is the lattice constant, and *V* is the unit cell volume.

Furthermore, the average crystallite size (*D*) decreased from 12.0 nm to 9.0 nm with increasing Ag content. Because Ag^+^ (1.26 Å) has a considerably larger ionic radius than host Mg^2+^ (0.72 Å), true lattice substitution would induce lattice expansion and shift the diffraction peaks toward lower 2*θ* angles. Consequently, the observed shift toward higher2*θ* values and unit cell contraction confirm that silver does not substitute for magnesium in the lattice. Instead, these structural changes are attributed to interfacial compressive stress and heterostructure formation at the Ag/MgO phase boundaries. The presence of surface-anchored metallic silver nanoparticles effectively pins the grain boundaries and inhibits the crystallite growth of the MgO matrix. The calculated structural parameters for all samples are summarized in Table 1.

**Table 1:** Microstructural parameters of pure MgO-NPs and Ag/MgO NCs

Sample	Structure	2*θ* of (200) (°)	Crystallite size (*D*, nm)	Lattice constant (*a*, Å)	Cell volume (*V*, Å^3^)
Pure MgO	Cubic	42.69	12.0	4.212	74.74
2% Ag/MgO	Cubic + Ag	42.75	11.5	4.208	74.53
4% Ag/MgO	Cubic + Ag	42.78	11.0	4.205	74.37
6% Ag/MgO	Cubic + Ag	42.81	10.2	4.201	74.16
8% Ag/MgO	Cubic + Ag	42.85	9.5	4.198	74.00
10% Ag/MgO	Cubic + Ag	42.91	9.0	4.194	73.29

Finally, the dislocation density *δ*, representing the defect concentration and structural quality of the doped lattice, was determined utilizing the following formulated eqn (7):^46^7
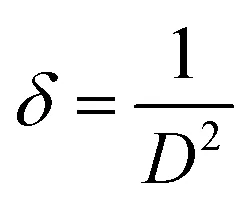


#### 3.2.2. Energy-dispersive X-ray (EDX) analysis

Energy-dispersive X-ray (EDX) spectroscopy confirmed the elemental composition and purity of the nanoparticles, showing clear peaks for Mg, O, and Ag, which verify the successful formation of Ag/MgO NCs. A minor carbon (C) peak, attributed to residual organic phytochemicals from the pepper seed extract used in synthesis, was also present. No other impurities were detected. Additionally, the Ag peak intensity increased with higher silver dopant concentrations (2% to 10%).

**Fig. 5:**
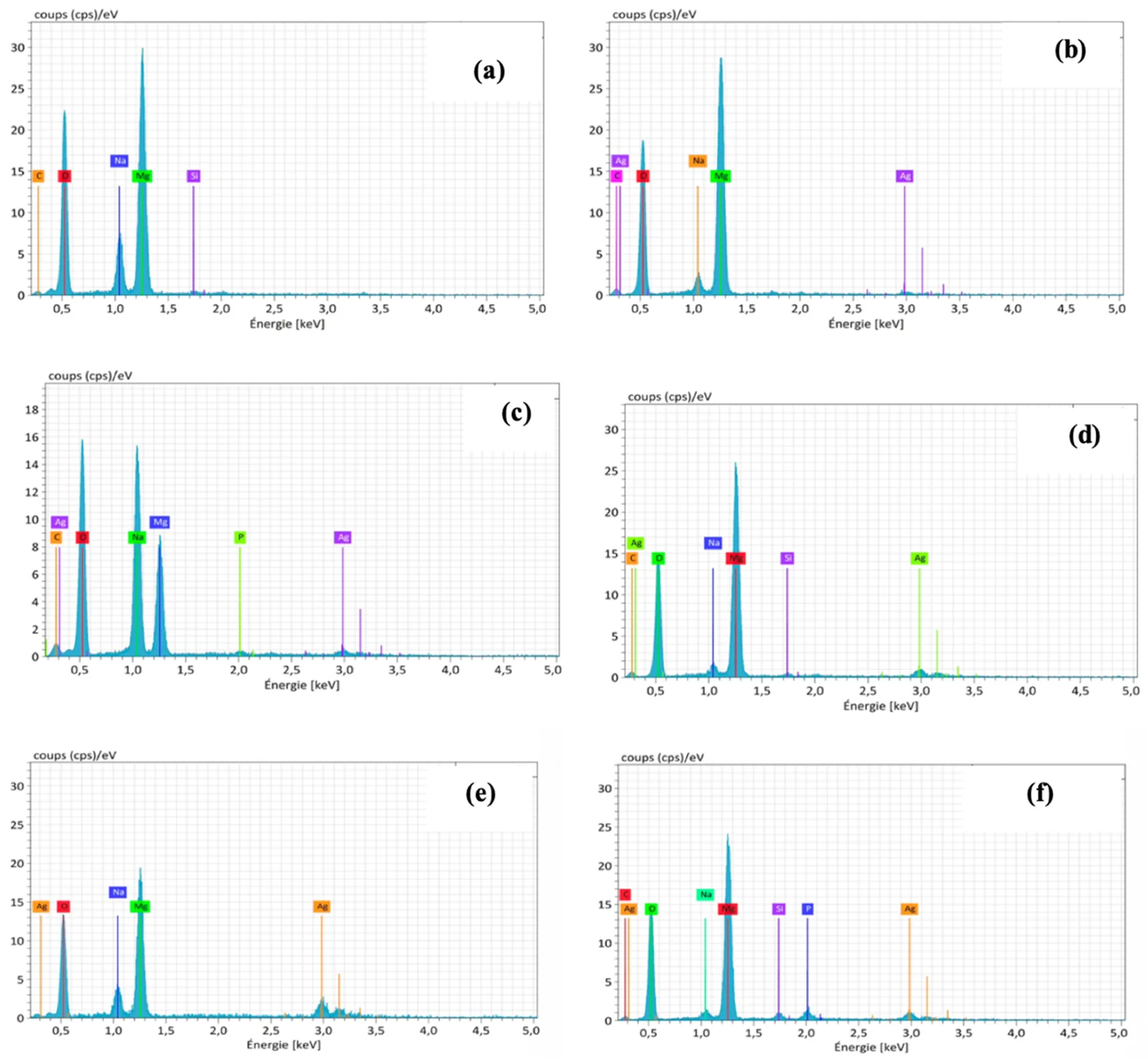
EDX spectra of (a) pure MgO and (b–f) Ag/MgO NCs at different Ag concentrations (2% to 10%).

The progressive increase in the intensity of the Ag peaks, relative to the Mg and O signals (Fig. 5), qualitatively confirms the successful incorporation of silver in proportion to the initial dopant concentrations.^47^ The consistent presence of these elemental signatures across various scanned regions suggests a homogeneous distribution of Ag nanoparticles within the MgO matrix.^48^ Furthermore, the high signal-to-noise ratio and the absence of extraneous peaks confirm the high chemical purity and crystallinity of the final nanostructures.

#### 3.2.3. Scanning electron microscopy (SEM)

**Fig. 6:**
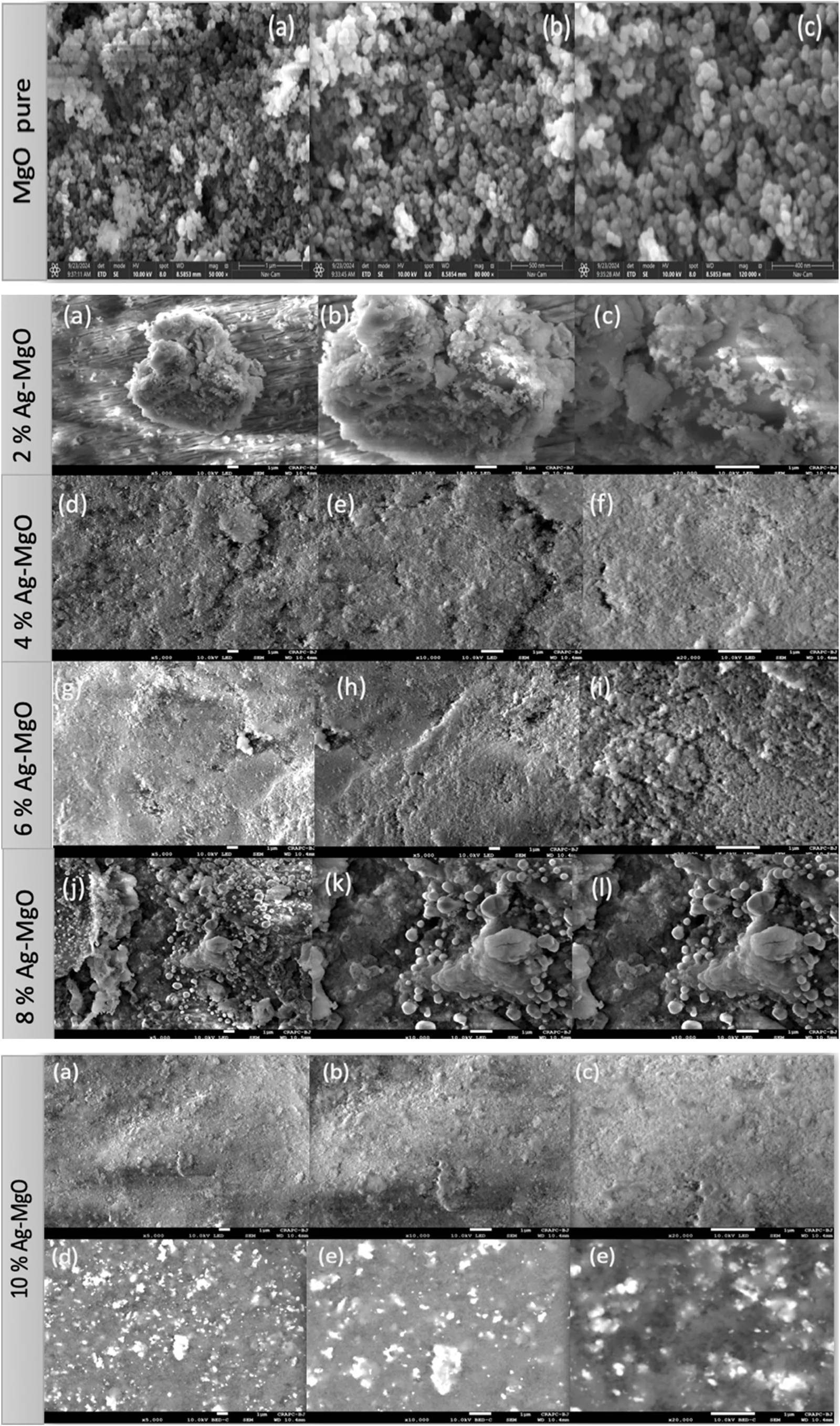
SEM analysis of the pure and Ag/MgO nanocomposite showcasing morphological changes with increasing silver content. (i) SEM micrographs of the pure MgO-NPs synthesized *via* the green route at different magnifications: (a) 50 000×, (b) 80 000×, and (c) 120 000×. (ii) SEM images of Ag-doped MgO at various silver concentrations and magnifications: (a–c) 2% Ag–MgO, (d–f) 4% Ag–MgO, (g–i) 6% Ag–MgO, and (j–l) 8% Ag–MgO. (iii) Surface morphology and phase contrast analysis of the 10% Ag–MgO sample: (a–c) secondary electron (SE) mode showing surface topography, and (d–f) backscattered electron (BSD) mode highlighting the distribution of Ag nanoparticles (bright spots) on the MgO matrix.

The surface morphology and particle distribution of the pure and Ag-doped MgO nanoparticles were examined using scanning electron microscopy (SEM), as displayed in Fig. 6. The micrographs of pristine MgO reveal a highly porous and granular structure consisting of nearly spherical and irregularly shaped agglomerates. This porous nature is characteristic of green synthesis, where phytochemicals from the pepper seed extract act as capping and stabilizing agents, influencing the nucleation and growth of the NPs.^49^

Upon the incorporation of silver (Ag), a noticeable change in surface texture is observed. The Ag/MgO samples exhibit a more compact and clustered morphology. As the dopant concentration increases from 2% to 8%, small, bright contrast spots appear on the surface of the MgO matrix, which are attributed to the presence of deposited Ag nanoparticles. The high surface area and porous network observed across all samples are beneficial for potential applications in photocatalysis and environmental remediation.^50^

SEM analyses showed that pure MgO nanoparticles have a granular, quasi-spherical, and highly agglomerated morphology, forming a porous network.^51^ Such agglomeration is typical for green-synthesized metal oxides using pepper seeds extract as a capping agent.

With 2–10% Ag doping, MgO's primary structure stays stable, but its surface changes. At 2–8% Ag, nanocomposite become more densely packed, indicating Ag clusters integrate into the MgO surface.^52^

The clearest evidence of successful doping comes from the 10% Ag–MgO sample. SE mode shows topography and grain boundaries,^53^ while BSD mode highlights chemical contrast, with silver's higher atomic number (*Z* = 47) producing bright white spots evenly distributed in the MgO matrix.^54^

#### 3.2.4. Fourier-transform infrared spectroscopy (FTIR)

**Fig. 7:**
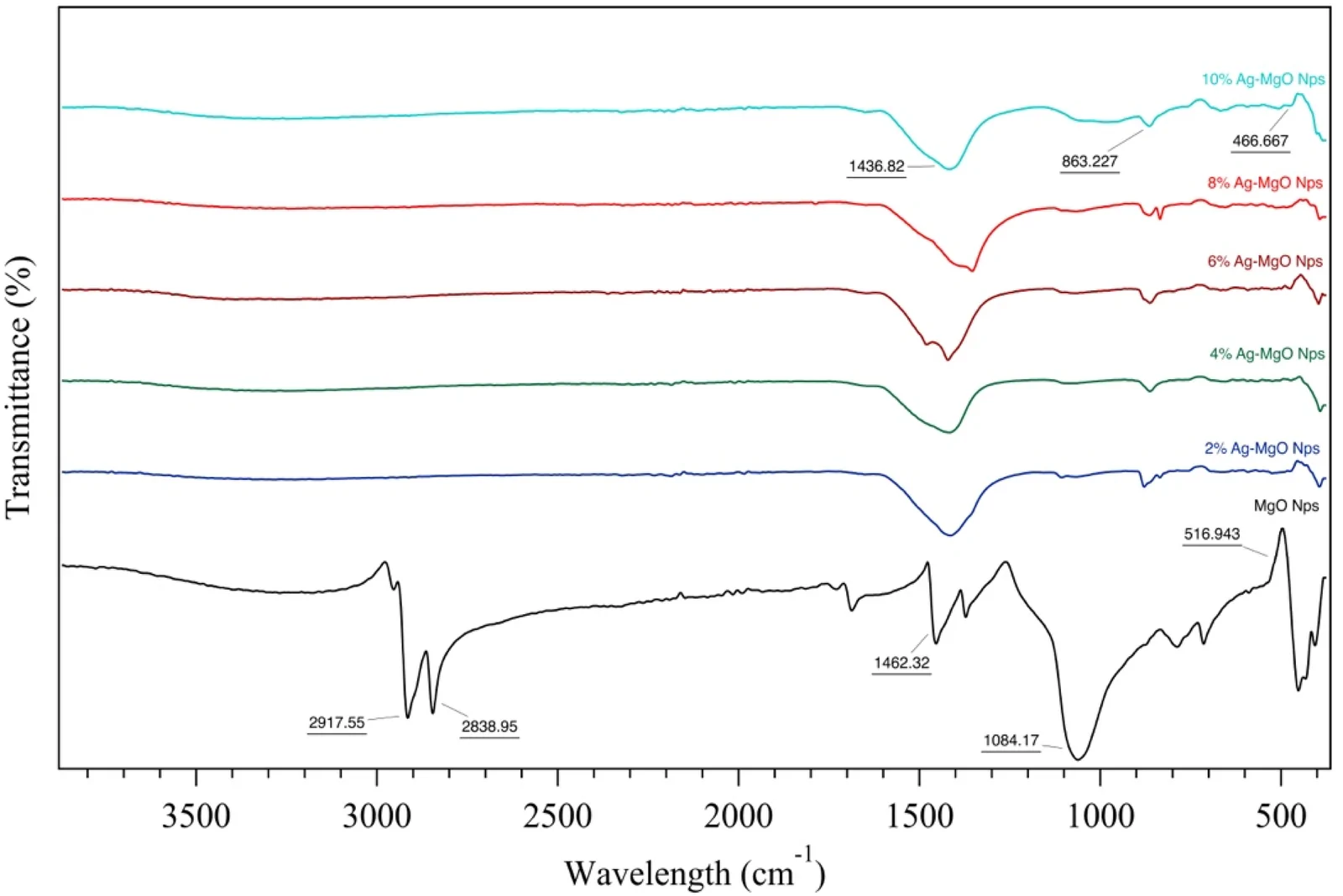
FT-IR spectra of pure and (2–10%) Ag/MgO NCs.

The chemical bonding and functional groups of the pure and Ag/MgO NCs were investigated using FTIR spectroscopy, as illustrated in Fig. 7. The spectra were recorded at room temperature across the wavenumber range of 400 to 4000 cm^−1^.

As illustrated in Fig. 7, the FTIR spectrum of pristine MgO nanoparticles (MgO-NPs) exhibits several intense characteristic bands. Notably, peaks at 2917.55 cm^−1^ and 2838.95 cm^−1^ correspond to symmetric and asymmetric C–H stretching vibrations of aliphatic groups,^55^ with high intensity indicating the presence of bioactive compounds from pepper seed extract capping the nanoparticle surface. Additional peaks at 1462.32 cm^−1^ (C–H bending) and 1084.17 cm^−1^ (C–O stretching) further confirm this interaction.^56^ The intense absorption band at 516.943 cm^−1^ reflects the Mg–O stretching vibration, validating the cubic MgO lattice formation.^57^ Upon doping with silver (2% to 10% Ag/MgO NCs), significant changes arise. Surface modifications are noted by the broadening and decreased intensity of organic peaks in the doped samples, indicating a reconfiguration of the organic layer due to the presence of silver nanoparticles.^58^ Shifts in peak locations are evident, with the C–H bending vibration shifting from 1462.32 cm^−1^ in the pure sample to 1436.82 cm^−1^ in the 10% Ag/MgO sample and a lattice-related band shift towards 863.227 cm^−1^. These changes imply strong Ag–O interactions and confirm the successful integration of silver into the MgO matrix.^47^ The Mg–O band remains at 466.667 cm^−1^ across all doped samples, highlighting that the structural integrity of the MgO host is preserved even with increasing silver concentrations.^58^ Overall, this FTIR analysis indicates that pepper seed extract plays a crucial role in a green synthesis pathway, reducing metal ions while providing a stabilizing organic shell for Ag/MgO nanostructures.

#### 3.2.5. Optical absorption properties (UV-vis DRS)

**Fig. 8:**
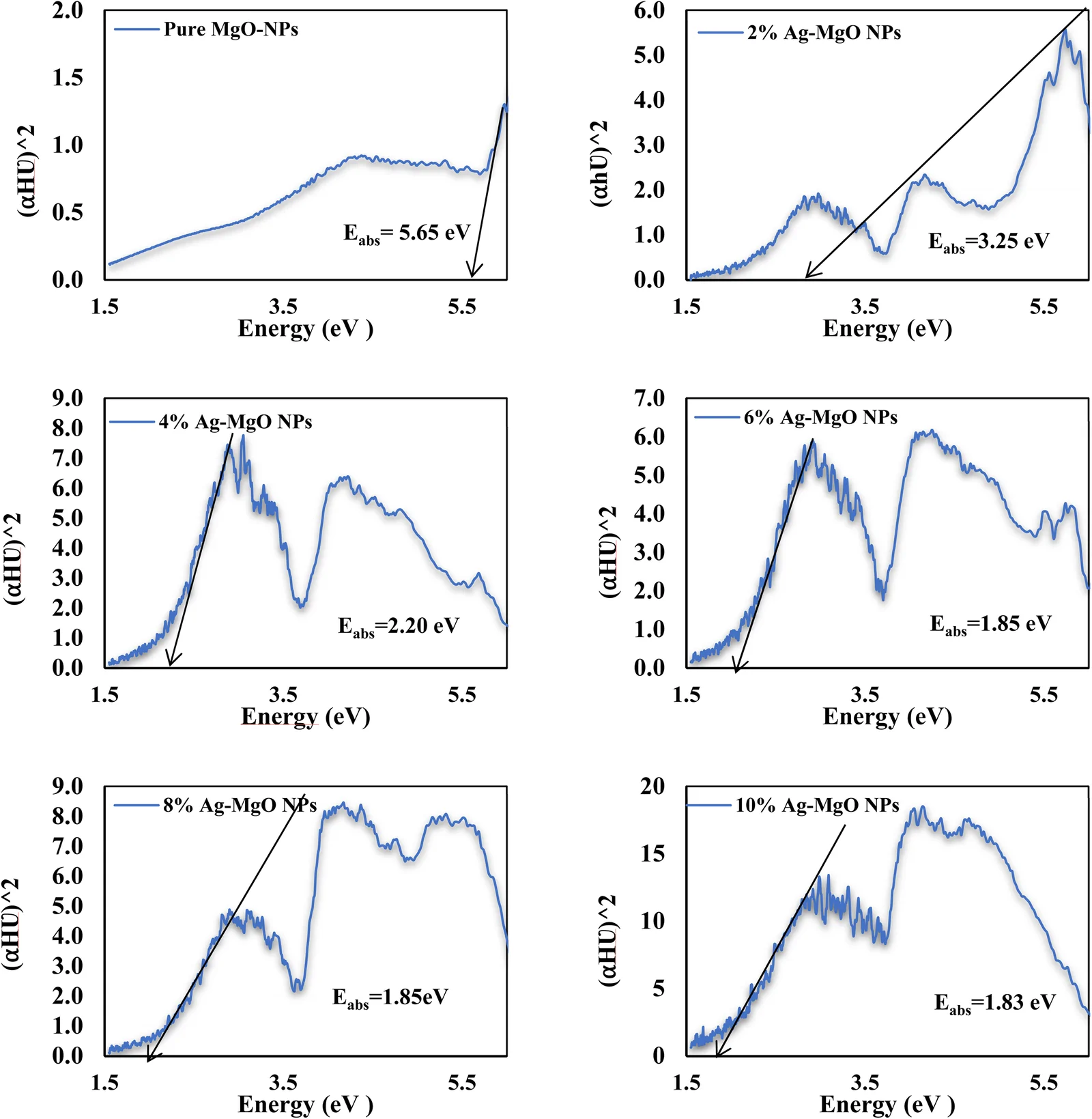
Transformed Kubelka–Munk plots of pristine MgO and Ag/MgO NCs highlighting the apparent visible-light absorption threshold (*E*_abs_) induced by Ag localized surface plasmon resonance (LSPR) and interfacial defect states.

The optical absorption properties of the pure and Ag/MgO NCs were investigated using UV-Vis diffuse reflectance spectroscopy (DRS). Fig. 8 Presents the acquired reflectance spectra alongside the corresponding Tauc plots, where the optical band gap (*E*_g_) was estimated by applying the Kubelka–Munk model and the Tauc equation for direct transitions eqn (8):

8 (*αhv*)^2^ = *A*(*hv* − *E*_g_)

As observed, pure MgO nanoparticles exhibit a distinct absorption edge in the deep UV region,^59^ corresponding to an inherently wide band gap of 5.65 eV. This high-energy threshold confirms the insulating nature of the pristine matrix and fundamentally restricts its optical activity to the ultraviolet spectrum.

The incorporation of silver (Ag) into the MgO matrix induces a pronounced redshift in the absorption edge, as evidenced by the narrowing of the optical band gap in the Tauc plots.

The apparent optical absorption threshold was reduced to 3.25 eV for 2% Ag loading and continued to decrease significantly to 2.20 eV for the 4% sample. As the Ag concentration increased to 6% and 8%, the values stabilized around 1.85 eV, eventually reaching a minimum of 1.83 eV for the 10% Ag–MgO NCs. This reduction is primarily attributed to the localized surface plasmon resonance (LSPR) of metallic Ag clusters along with the creation of new intermediate energy levels (impurity and defect states) at the Ag/MgO interface, which facilitate electronic transitions at lower energies.^52^ These results confirm that silver doping effectively tunes the MgO host for visible-light-driven applications.^60^

### 3.3. Photocatalytic performance

The photocatalytic efficiency of MgO-NPs and Ag/MgO NCs is highly dependent on operational conditions, including initial pH, catalyst dosage, and initial DCF concentration.

#### 3.3.1. Effect of Ag dopant concentration

**Fig. 9:**
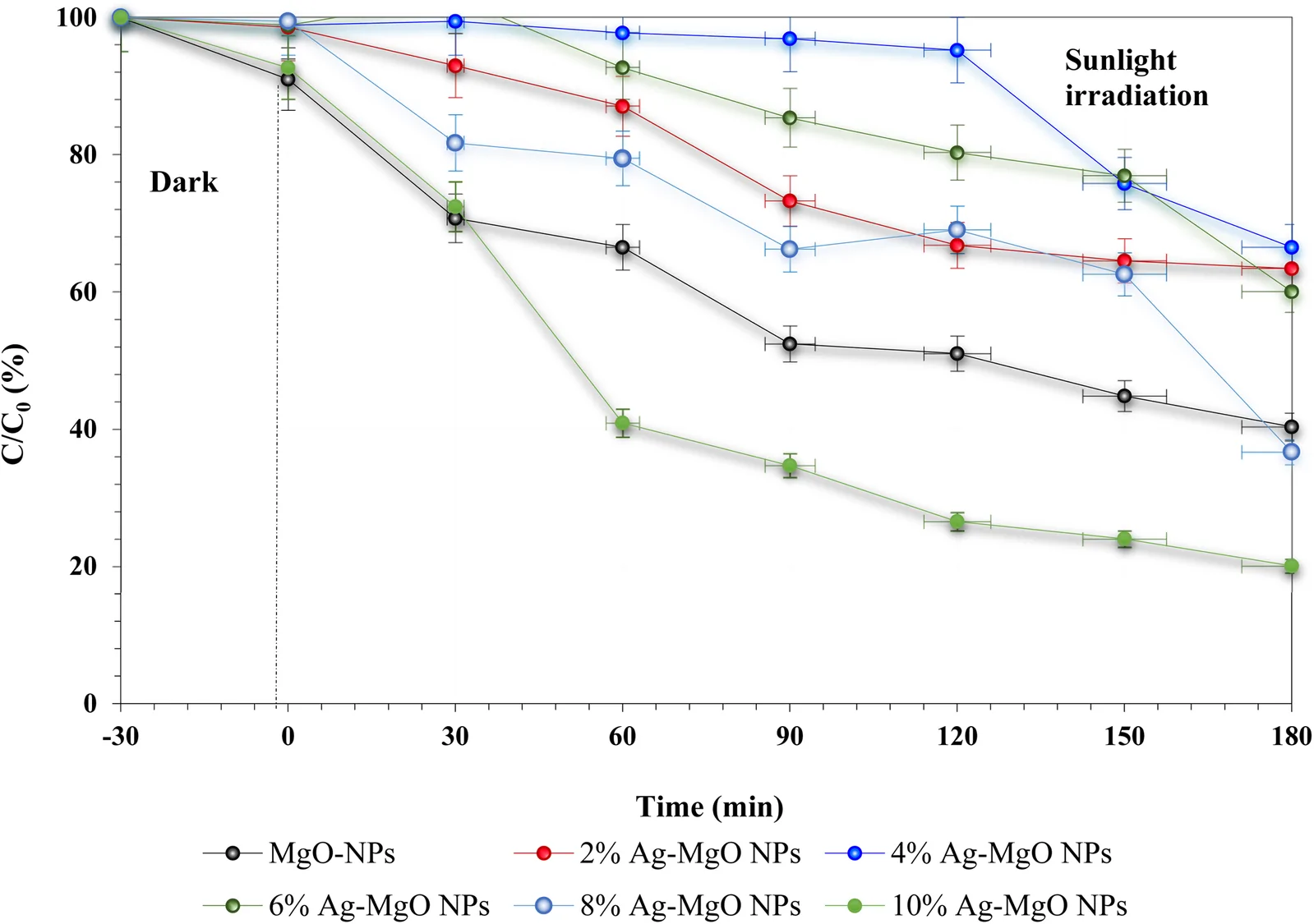
Ag dopant concentration effect (2% to 10% (w/w): *m* = 1g L^−1^, pH = 5.48, [DCF] = 10 ppm, *T* = 27.5 °C.

The influence of silver (Ag) loading on the photocatalytic activity of MgO-NPs was evaluated by varying the dopant concentration from 2% to 10% (w/w). As shown in Fig. 9, the degradation efficiency of diclofenac (DCF) was significantly improved with increasing silver content under sunlight irradiation.

As illustrated in Fig. 9, the photocatalytic activity of Ag/MgO strongly depends on the Ag loading. At 2 wt% Ag, the photocatalytic performance is lower than that of pristine MgO, reaching a final normalized concentration (*C*/*C*_0_) of approximately 0.63 after 180 min of irradiation. A similar behavior is observed for the 4 wt% and 6 wt% Ag-loaded samples, which exhibit slower degradation kinetics, with the 4 wt% sample showing the lowest photocatalytic efficiency. Increasing the Ag loading to 8 wt% improves the degradation performance, resulting in activity comparable to that of pure MgO. The highest photocatalytic efficiency is achieved with the 10 wt% Ag/MgO photocatalyst, which outperforms all other samples by reducing the final normalized concentration to *C*/*C*_0_ ≈ 0.20, indicating that this Ag loading provides the optimum balance between charge separation and the availability of active surface sites for efficient diclofenac degradation.

#### 3.3.2. Effect of initial solution pH

**Fig. 10:**
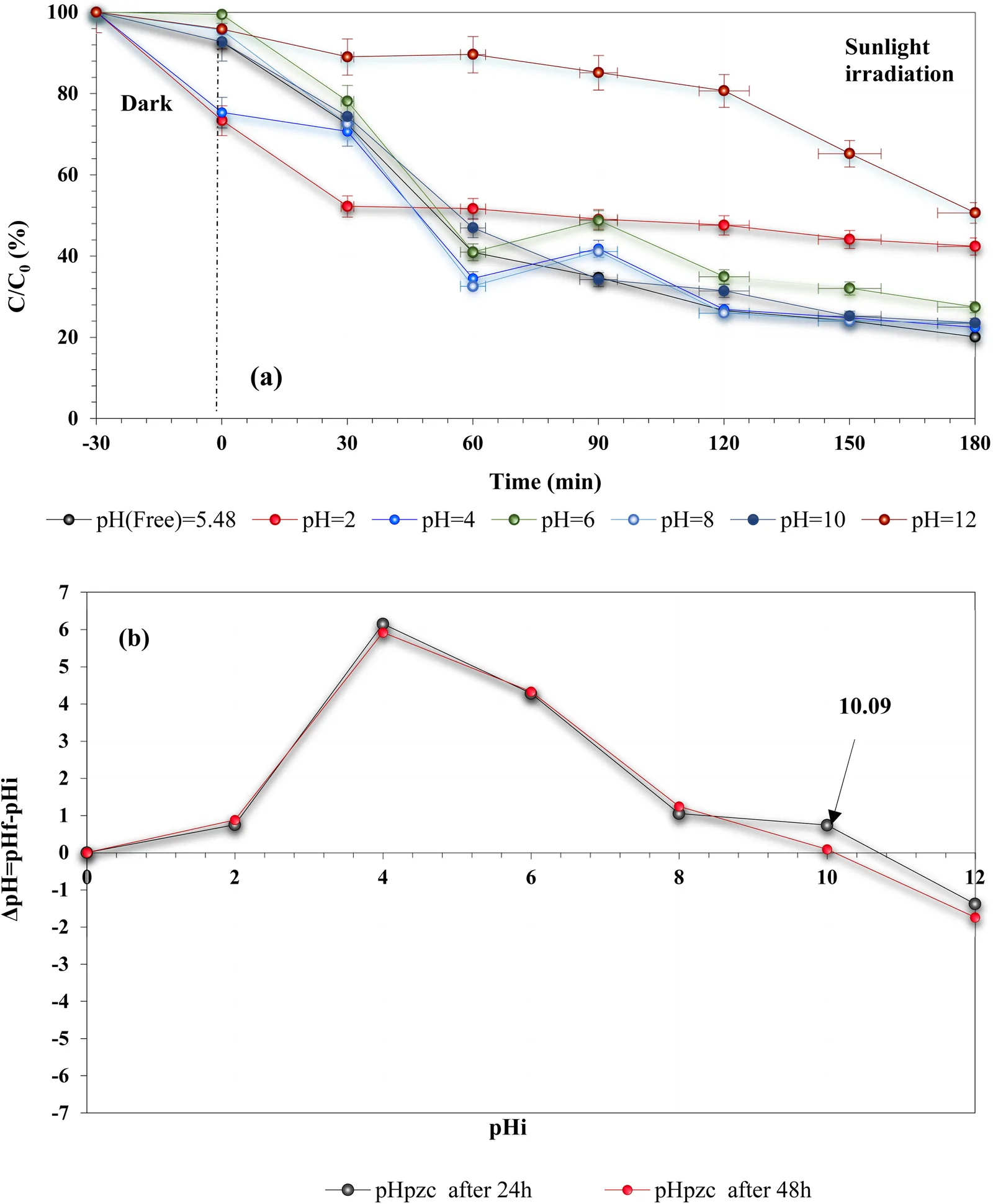
Effect of pH on photocatalytic efficiency (a) and point of zero charge (pH_P*Z*C_) analysis of 10% Ag/MgO NCs (b): *m* = 1g L^−1^, [DCF] = 10 ppm, *T* = 27.5 °C.

In this study, the influence of initial solution pH was evaluated on the removal efficiency of Diclofenac (DCF). The experimental findings illustrated in Fig. 10(a) indicate that the maximum degradation efficiency occurs at natural pH (5.48) and pH 6 after 180 minutes of sunlight irradiation. In contrast, a significant reduction in performance is observed at pH 12, where the efficiency drops to approximately 49%. The pH of the solution is a critical parameter in heterogeneous photocatalysis, as it governs the surface charge of the 10% Ag/MgO NCs, the degree of catalyst aggregation, and the potential for generating reactive oxygen species like hydroxyl radicals.

The observed changes in photocatalytic efficiency are primarily driven by electrostatic interactions between the catalyst surface and the DCF molecules. As shown in Fig. 10(b), the point of zero charge (pH_P*Z*C_) of the synthesized 10% Ag/MgO NCs was determined to be 10.09. This value is a crucial threshold: when the solution pH is below 10.09, the catalyst surface becomes protonated and carries a positive charge, which facilitates the electrostatic attraction of anionic pollutants like DCF. However, when the pH exceeds 10.09, the surface becomes deprotonated and negatively charged, resulting in electrostatic repulsion and the observed decrease in catalytic activity. This surface charging behavior is described by the following equilibrium equations:

• pH < pH_P*Z*C_: the surface is protonated and carries a positive charge eqn (9).

9 MgOH + H^+^ → MgOH_2_^+^

• pH > pH_P*Z*C_: the surface is deprotonated and carries a negative charge eqn (10).

10 MgOH + OH^−^ → MgO^−^ + H_2_O

#### 3.3.3. Effect of catalyst dosage

**Fig. 11:**
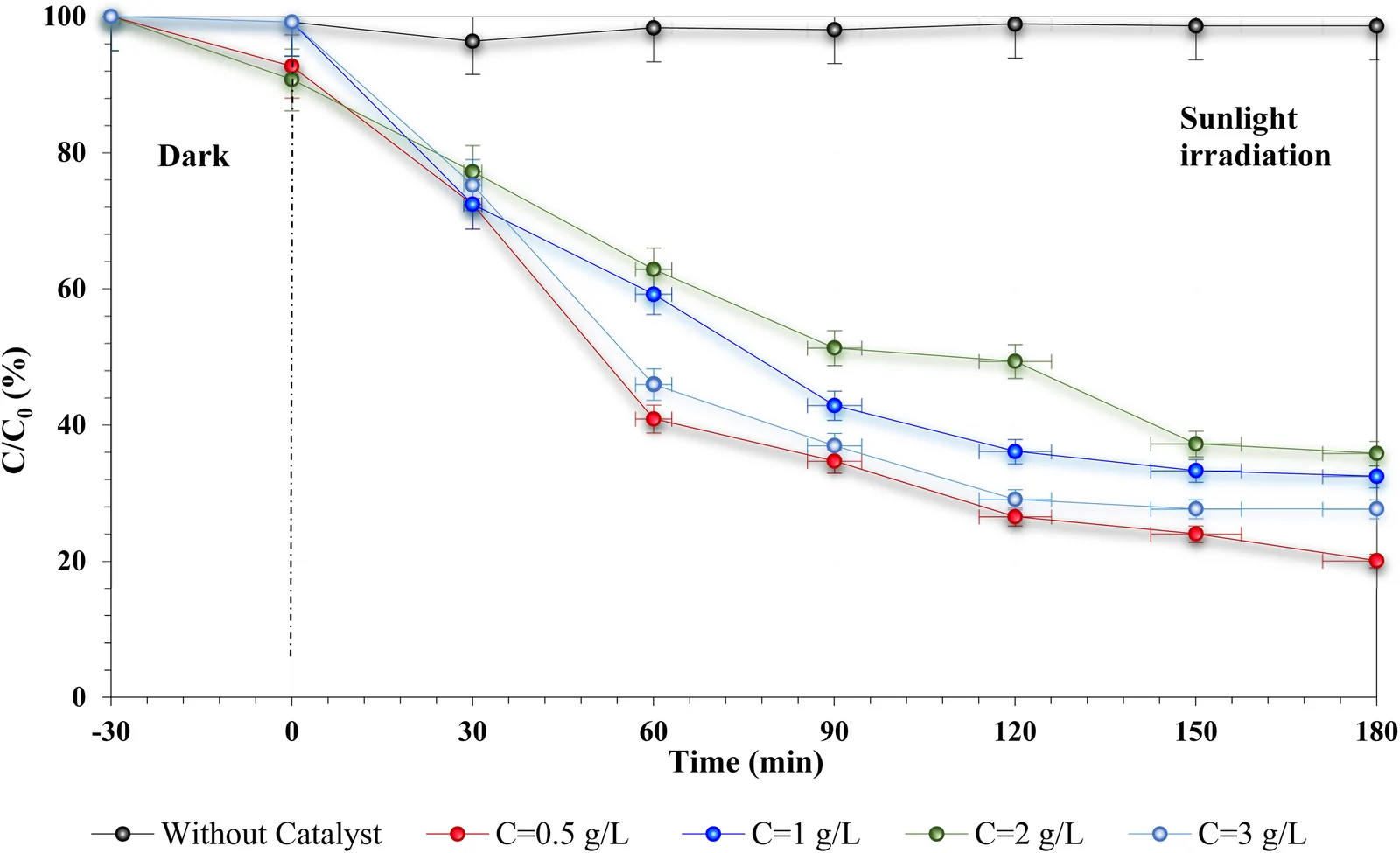
Catalyst dosage effect: pH = 5.48, [DCF] = 10 ppm, *T* = 27.5 °C.

The influence of catalyst dosage on the photodegradation kinetics of DCF was investigated by varying the amount of 10% Ag/MgO NCs from 0.5 g L^−1^ to 3 g L^−1^ under 180 minutes of sunlight irradiation (Fig. 11). Prior to irradiation, the suspension was stirred in the dark for 30 minutes to establish adsorption–desorption equilibrium.

The photocatalytic degradation efficiency strongly depended on the catalyst dosage, with the highest removal efficiency (approximately 80%) achieved at the lowest catalyst loading of 0.5 g L^−1^ (Fig. 11). Increasing the catalyst dosage to 2 and 3 g L^−1^ resulted in a noticeable decline in degradation efficiency, reaching approximately 64% and 68%, respectively.

These results indicate that a catalyst dosage of 0.5 g L^−1^ provides the optimum balance between the availability of active surface sites and efficient light utilization. Although increasing the catalyst dosage theoretically increases the number of active sites available for the generation of reactive oxygen species (ROS), excessive amounts of catalyst also increase the turbidity of the suspension, thereby reducing light penetration into the reaction medium.^61^ This phenomenon, commonly referred to as the “light-screening” or “shielding” effect, promotes light scattering and limits the photoactivation of catalyst particles located in the inner regions of the suspension.^62^ As a result, fewer photogenerated electron–hole (e^−^/h^+^) pairs are produced, leading to a lower generation of reactive species and, consequently, a decrease in the overall photocatalytic degradation.^63^

#### 3.3.4. Effect of initial DCF concentration

The outcomes demonstrate that the initial DCF concentration is a decisive factor in determining the overall efficiency of the photocatalytic process. In this study, DCF concentrations ranging from 5 to 20 ppm were evaluated under 180 min of sunlight irradiation.

**Fig. 12:**
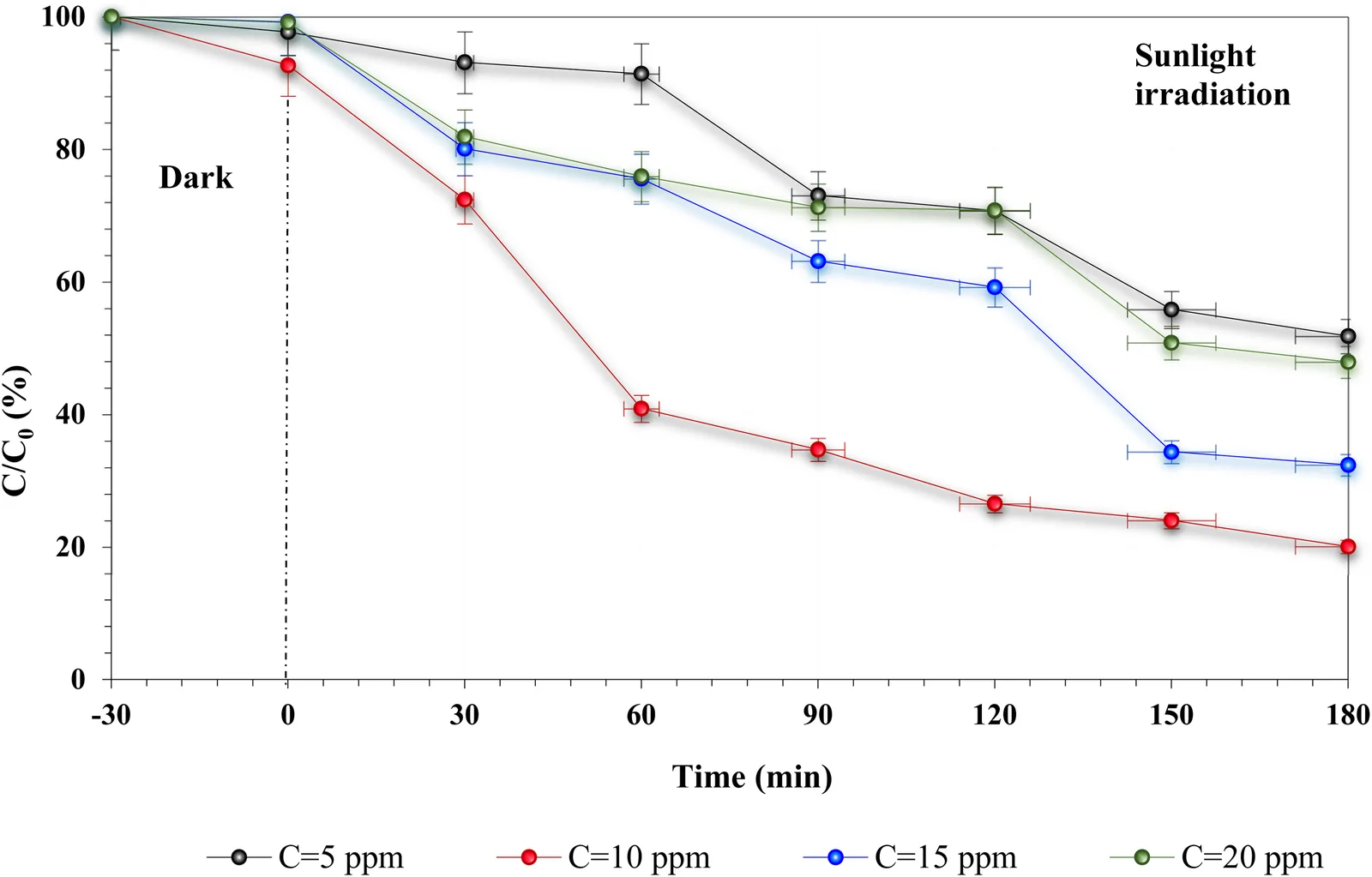
Effect of DCF initial concentration on DCF removal efficiency: *m* = 0.5g L^−1^, pH = 5.48, *T* = 27.5 °C.

The photocatalytic degradation efficiency was strongly influenced by the initial diclofenac concentration (Fig. 12). The highest removal efficiency (approximately 80%) was obtained at an initial concentration of 10 ppm. In contrast, both the lowest concentration (5 ppm) and the higher concentrations (15 and 20 ppm) resulted in lower degradation efficiencies, ranging from approximately 48% to 52%.

The superior performance observed at 10 ppm indicates that this concentration provides an optimum balance between pollutant availability and the generation of reactive oxygen species (ROS). At higher DCF concentrations, the photocatalytic efficiency decreases because the catalyst surface becomes progressively saturated with adsorbed DCF molecules, thereby reducing the number of available active sites for ROS formation.^64^ In addition, the increased optical density of the solution produces an inner filter (light-shielding) effect, which attenuates sunlight penetration and limits the generation of hydroxyl radicals (˙OH), resulting in slower degradation kinetics.^65^

At the lowest concentration (5 ppm), the reduced degradation efficiency can be attributed to the lower probability of interactions between DCF molecules and the photogenerated reactive species, leading to less efficient utilization of the generated ROS.^66^ Furthermore, the gradual accumulation of oxidation intermediates may compete with the parent DCF molecules for active sites and reactive radicals, thereby contributing to the observed decrease in degradation efficiency.^67^

#### 3.3.5. Effect of ionic strength

**Fig. 13:**
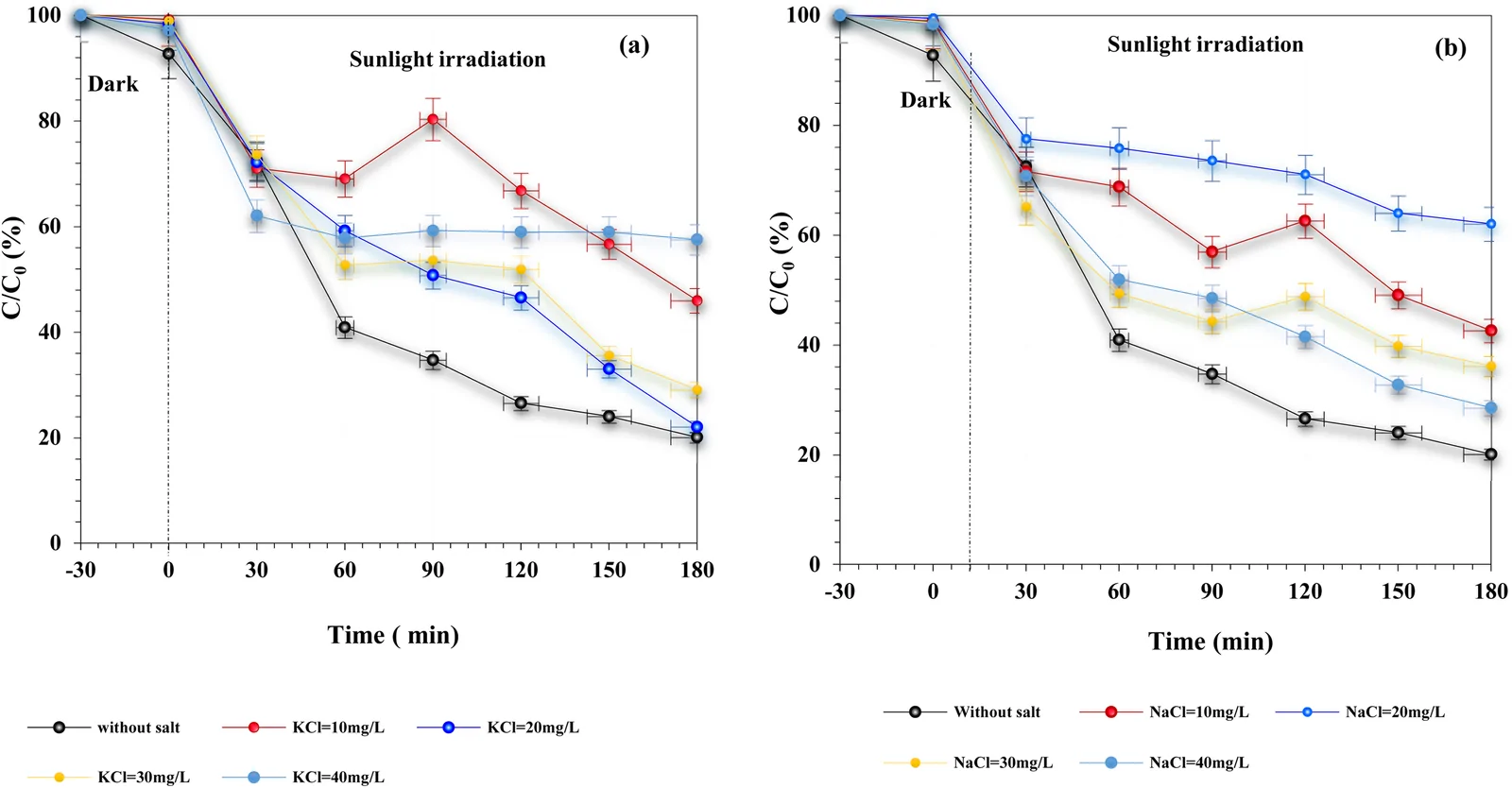
Effect of the ionic strength of (a) KCl and (b) NaCl on DCF removal efficiency: *m* = 0.5g L^−1^, pH = 5.48, *T* = 27.5 °C.

The influence of ionic strength on the photodegradation kinetics of DCF (10 ppm) was investigated by adding inorganic salts (KCl and NaCl) at concentrations ranging from 10 mg L^−1^ to 40 mg L^−1^ under 180 min of sunlight irradiation (Fig. 13). Prior to irradiation, the suspension was stirred in the dark for 30 min to establish adsorption–desorption equilibrium.

The photocatalytic degradation of diclofenac was markedly affected by the presence of inorganic salts (Fig. 13). The highest degradation efficiency was obtained in the absence of added salt, reaching a final normalized concentration of *C*/*C*_0_ ≈ 0.20 (corresponding to approximately 80% removal). In contrast, the addition of either KCl (Fig. 13(a)) or NaCl (Fig. 13(b)) resulted in a progressive decline in photocatalytic performance over the entire concentration range investigated.

The inhibitory effect of these salts can be attributed to several factors. First, chloride ions (Cl^−^) act as efficient scavengers of highly reactive hydroxyl radicals (˙OH), converting them into less reactive chlorine-containing species with lower oxidation potentials, thereby reducing the availability of oxidative radicals responsible for diclofenac degradation.^68,69^ In addition, the coexistence of Na^+^, K^+^, and Cl^−^ ions compete with DCF molecules for adsorption onto the active sites of the 10% Ag/MgO photocatalyst, leading to a lower adsorption capacity and reduced photocatalytic efficiency. Furthermore, increasing the ionic strength compresses the electrical double layer surrounding the catalyst particles, resulting in an electrostatic screening effect that weakens the interaction between DCF molecules and the catalyst surface, thereby further limiting the degradation process.^70^

#### 3.3.6. Comparative analysis of irradiation sources: natural sunlight *vs.* artificial light

**Fig. 14:**
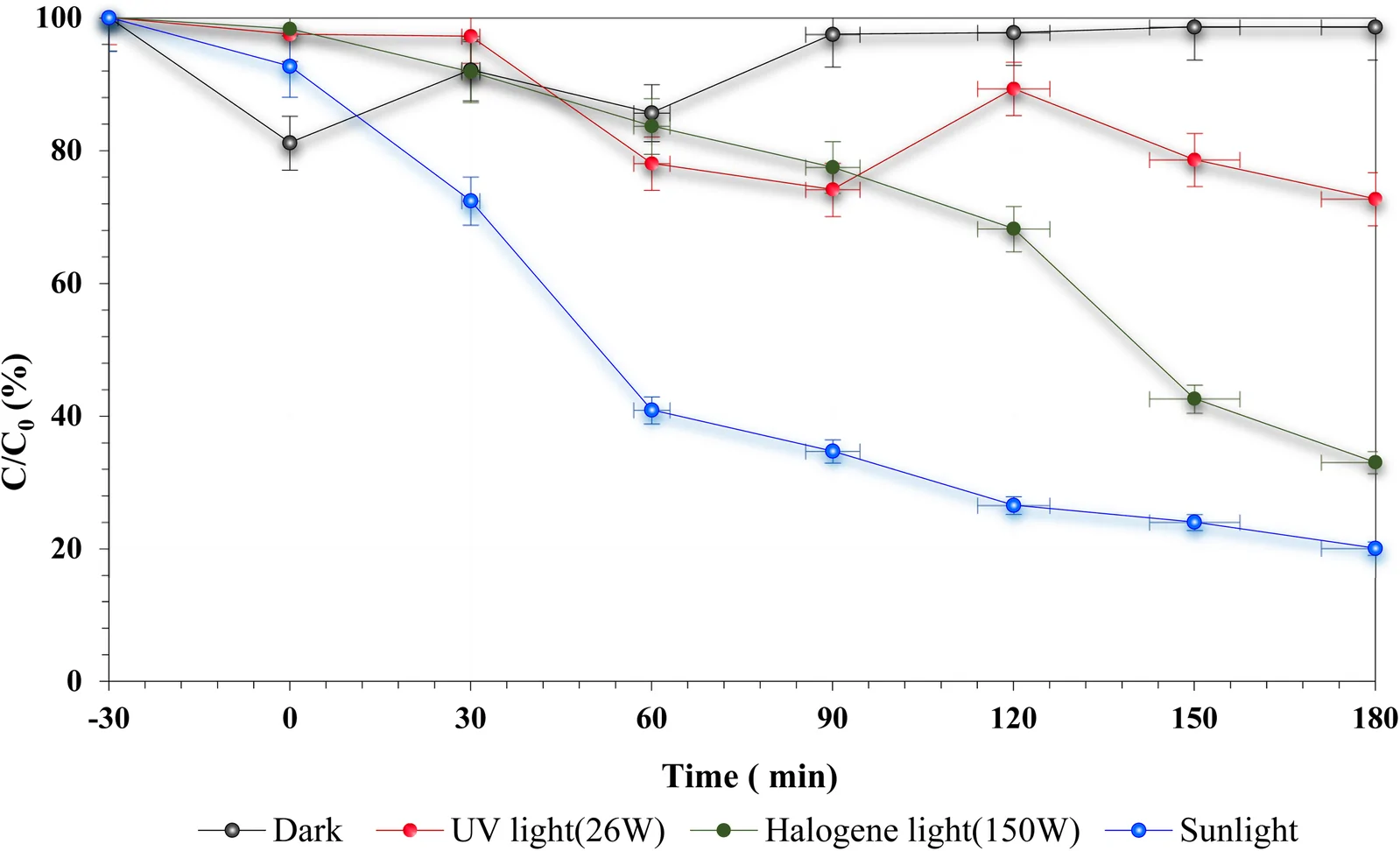
Comparative photocatalytic degradation of DCF using the 10%Ag/MgO NCs under natural sunlight, artificial UV light (26 W), and Halogen light (150 W).

To validate the solar-driven efficiency of the synthesized 10% Ag/MgO NCs, comparative experiments were conducted under various illumination conditions, as illustrated in Fig. 14. The results demonstrate that natural sunlight is the most effective and sustainable choice for the degradation process.

The photocatalytic degradation of diclofenac was strongly influenced by the irradiation source (Fig. 14). Natural sunlight irradiation exhibited the highest photocatalytic activity, showing markedly faster degradation kinetics than the artificial light sources investigated. During the first 60 min of irradiation, sunlight reduced the normalized diclofenac concentration to *C*/*C*_0_ ≈ 0.40, whereas the 150 W halogen lamp and the 26 W UV lamp achieved only *C*/*C*_0_ ≈ 0.80 and *C*/*C*_0_ ≈ 0.90, respectively.

The superior performance under sunlight can be attributed to the enhanced visible-light absorption of the 10% Ag/MgO nanoparticles resulting from Ag incorporation and the associated narrowing of the optical band gap. These modifications promote more efficient utilization of the solar spectrum and facilitate the generation of photogenerated charge carriers under natural irradiation conditions. The results demonstrate that the optimized Ag/MgO photocatalyst can operate effectively under direct sunlight without the need for high-power artificial irradiation, highlighting its potential for energy-efficient and large-scale solar-driven wastewater treatment applications. A comparison of the DCF degradation efficiency achieved in this study with other previously reported catalysts is summarized in Table 2.

**Table 2:** Comparison of degradation efficiency for various samples on DCF pollutant

Samples	Concentration (ppm)	Source of light	Time (min)	Efficiency	Ref.
Ag-BiOI-rGO	10 ppm	Halogen (300 W)	80 min	99.0%	80
MoO_3_@ZrO_2_	10 ppm	LED (500 W)	150 min	90.94%	81
CeO_2_/g-C_3_N_4_	10 ppm	LED (200 W)	180 min	91.4%	82
g-C_3_N_4_/TiO_2_	20 ppm	Sunlight irradiation	240 min	73.0%	83
10% Ag/MgO NCs	10 ppm	Sunlight irradiation	180 min	80.0%	This study

### 3.4. Scavenging experiment and mechanism of the photodegradation

The photocatalytic degradation of diclofenac sodium is accompanied by the redox reactions, which are induced by the generation of free radicals such as hydroxyl radicals (˙OH), superoxide radicals (˙O_2_^−^), and holes (h^+^) through the photocatalytic process. The scavenging studies against the diclofenac sodium were carried out to investigate the role of these radicals in the photodegradation of diclofenac sodium using MgO-NPs and Ag/MgO NCs. Different scavengers such as *tert*-butanol (T-BuOH), benzoquinone (BQ), ammonium oxalate (Na_2_C_2_O_4_), and silver nitrate (AgNO_3_) were used as the quenchers of ˙OH, ˙O_2_^−^, h^+^, and e^−^, respectively.^71^ The results demonstrated that the addition of benzoquinone (BQ) and *tert*-butanol (T-BuOH) led to a significant decrease in the degradation rate, indicating that ˙O_2_^−^ and ˙OH are the dominant active species in the sunlight-driven process.^72^ The role of h^+^ was also vital, as ammonium oxalate partially suppressed DCF removal, suggesting direct oxidation by holes on the catalyst surface.^73^ Conversely, the addition of AgNO_3_ as an electron scavenger often enhances performance by reducing e^−^/h^+^ recombination, further proving the essential role of charge separation.^74^

**Fig. 15:**
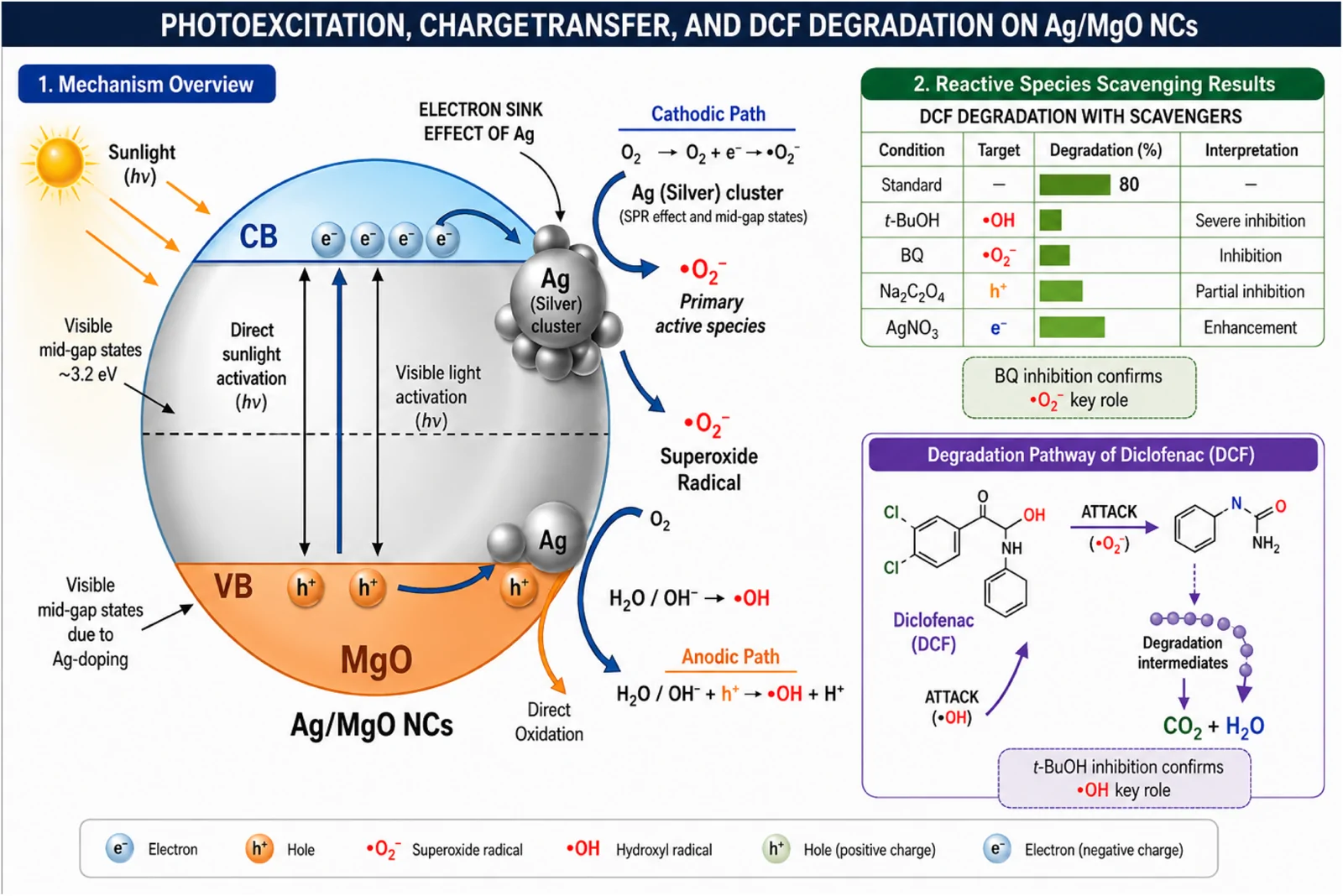
A proposed mechanism diagram of DCF photodegradation on 10% Ag/MgO NCs.

**Fig. 16:**
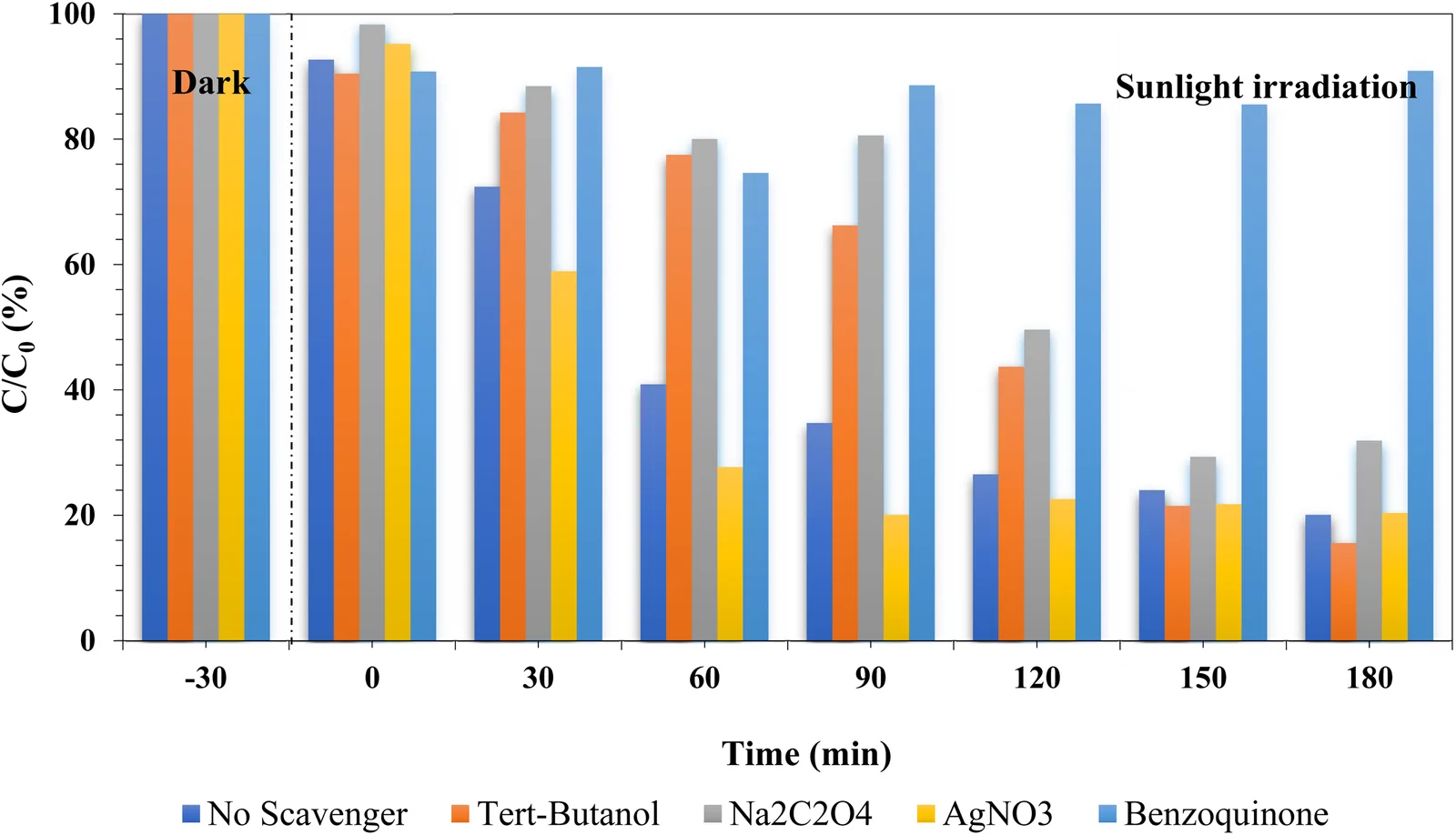
Radical scavenging test over 10% Ag/MgO NCs.

The possible mechanism of the enhanced photocatalytic activity of 10% Ag/MgO NCs is proposed in Fig. 15. The improved efficiency of 10%Ag/MgO NCs compared to pure MgO is attributed to a synergistic effect. While MgO NPs has a wide bandgap, Ag-doping introduces mid-gap states that allow for visible light activation. Upon direct sunlight irradiation, electrons in the valence band (VB) of MgO are excited to the conduction band (CB), leaving an equal number of holes in the valence band (eqn (11)) (Fig. 16).^75^

The metallic Ag dopant acts as an electron sink, effectively trapping the excited conduction band electrons and preventing their rapid recombination with VB holes. These trapped electrons react with dissolved oxygen (O_2_) to generate superoxide radicals (˙O_2_^−^) (eqn (12)).^76^ Simultaneously, the generated holes in the VB migrate to the catalyst surface to oxidize water molecules or OH− ions into hydroxyl radicals (˙OH) (eqn (13))^77^ or directly attack and oxidize the DCF molecules (eqn (14)).^78^ These reactive oxygen species (ROS), along with direct hole oxidation, break down the stable DCF into intermediate products, ultimately mineralizing them into CO_2_ and H_2_O.^79^

11 MgO + *hv* → e^−^ + h^+^

12 e^−^ + O_2_ → O_2_˙^−^

13 h^+^ + H_2_O →˙OH + H^+^

14 (h^+^,O_2_˙^−^,˙OH) + organic (DCF) → CO_2_ + H_2_O

### 3.5. Mineralization and total organic carbon (TOC) evaluation

To determine whether the solar photocatalytic treatment achieves true mineralization of diclofenac rather than partial chromophore destruction, total organic carbon (TOC) removal was systematically investigated considering the optimized 10 wt% Ag/MgO NCs under natural sunlight irradiation. While UV-Vis absorbance monitoring tracks the cleavage of the central secondary amine bridge and aromatic conjugated chromophores, TOC measurements quantify the total organic carbon content remaining in the aqueous solution, providing direct insight into complete conversion toward harmless end-products (CO_2_, H2O, and inorganic ions) (Table 3).

**Table 3:** DCF degradation and TOC removal efficiencies at various irradiation times

Irradiation time (min)	Residual DCF (mg L^−1^)	*C*/*C*_0_ (%)	DCF degradation efficiency (%)	Residual TOC (mg L^−1^)	TOC removal (mineralisation) (%)
−30	10.543	100.000	0.00	20.510	0.00
0	9.772	92.68	7.32	20.140	1.80
60	4.312	40.90	59.10	14.260	30.47
120	2.798	26.54	73.46	10.510	48.708
180	2.116	20.07	80.00	9.530	53.54

### 3.6. Reaction kinetics

It is important to understand the reaction kinetics for the degradation of diclofenac sodium. The photodegradation reaction follows pseudo-first-order kinetics regarding the pollutant concentration, provided the sufficient light and suitable catalytic sites.^84^

The kinetic model is expressed by the following linear equation (eqn (15)):^85^15
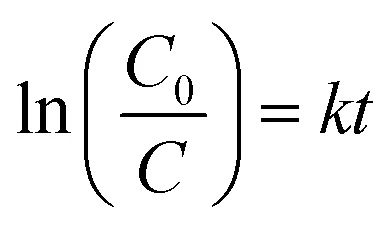
where: *C*_0_ is the initial concentration of the pollutant. *C* is the concentration at time *t*. *k* is the apparent pseudo-first-order rate constant (min^−1^).

**Fig. 17:**
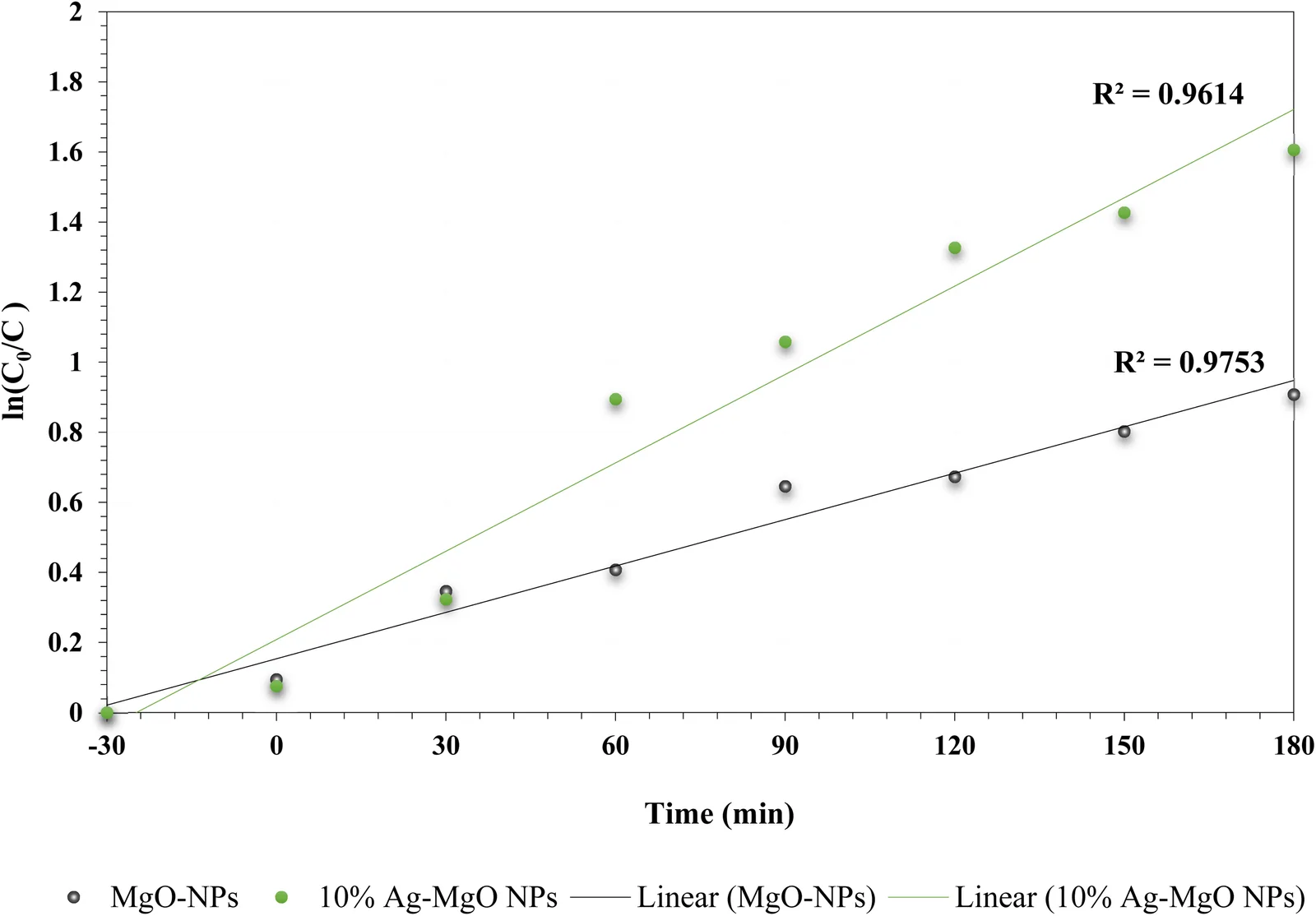
Photodegradation kinetics of MgO-NPs and 10% Ag–MgO NPs.

This kinetic expression is derived from the Langmuir–Hinshelwood model, which simplifies to a pseudo-first-order rate law at low initial pollutant concentrations (10 ppm) where surface active sites remain in relative excess. Alternative kinetic models (such as pseudo-second-order) were also tested but yielded lower correlation coefficients (*R*^2^ < 0.91), confirming that the solar photocatalytic degradation process was accurately described by apparent rate constants (*k*) of 0.0046 min^−1^ for pure MgO-NPs and 0.0084 min^−1^ for the optimized 10% Ag–MgO nanocomposite, as illustrated in Fig. 17.

Fig. 17. Shows the good linear fit of the ln(*C*_0_/*C*) *vs.* irradiation time with *R*^2^ values of 0.975 for MgO-NPs and 0.961 for 10% Ag–MgO NPs. The *R*^2^ values approaching 1 indicated that diclofenac degradation followed pseudo-first-order kinetics. The degradation rate constants obtained from the slope of linear plots were 0.0046 min^−1^ and 0.0084 min^−1^ for pure MgO-NPs and 10% Ag/MgO NCs respectively in Table 4. The higher value of the degradation rate constant for 10% Ag/MgO NCs demonstrates that the incorporation of silver significantly accelerates the degradation rate of diclofenac sodium.

**Table 4:** The photodegradation reaction conditions, correlation coefficient (*R*^2^), and rate constant for the degradation of diclofenac sodium

Photocatalysts	Experimental condition			Pseudo-first order kinetics	
	Conc. (ppm)	Dose (g L^−1^)	Time (min)	*k* (min^−1^)	*R* ^2^
MgO-NPs	10	0.5	180	0.0046	0.975
10% Ag/MgO NCs	10	0.5	180	0.0084	0.961

### 3.7. DT_LSBoost model optimization and predictive performance

The predictive capability of ensemble learning models is largely governed by the selection of their hyperparameters, which determine both the complexity of the base learners and the convergence behavior of the boosting procedure. In this study, the EEFO algorithm was adopted to automatically identify the optimal DT_LSBoost configuration. The optimization process was conducted using a population of 100 candidate solutions that evolved over 200 iterations, allowing an extensive exploration of the search space while ensuring convergence toward the most suitable parameter combination. The resulting optimal hyperparameter values are presented in Table 5.

Performances of the best model of DT_LSBOOST

**Table:** 

Min leaf size: 1
Learn rate: 0.1
Surrogate: all
Min parent size: 2
Number of learng cycles: 28
Max number splits: 150

**Table:** 

Coefficients of correlation				RMSE			
Train	Test	VAL	ALL	Train	Test	VAL	ALL
0.9999	0.9999	0.9999	0.9999	0.0012	0.0013	0.0011	0.0012

The optimization procedure identified a compact yet highly flexible ensemble architecture. A minimum leaf size of one observation and a minimum parent node size of two observations enabled each regression tree to preserve fine-scale information contained within the experimental dataset. Simultaneously, allowing a maximum of 150 tree splits provided sufficient structural flexibility to model the nonlinear behavior of the photocatalytic degradation process without introducing unnecessary complexity. The boosting component converged to an optimal learning rate of 0.1, ensuring that each successive learner contributed progressively to the correction of residual errors. Moreover, the optimization indicated that 28 boosting iterations were sufficient to maximize predictive accuracy, while additional learning cycles produced no significant performance improvement. The use of surrogate splits for all predictor variables further increased the robustness of the model by maintaining reliable tree construction whenever alternative splitting variables were required.

Following hyperparameter optimization, the predictive performance of the DT_LSBoost model was evaluated using the *R* and the RMSE. The statistical indicators obtained for the training, testing, validation, and complete datasets are summarized in Table 4. Remarkably, the model achieved an identical correlation coefficient of 0.9999 for all datasets, indicating an almost perfect correspondence between the predicted and measured values. Such exceptionally high agreement demonstrates that the optimized ensemble accurately captured the complex interactions linking the operating conditions to the photocatalytic degradation efficiency.

The prediction errors remained consistently low across all evaluation stages. RMSE values of 0.0012, 0.0013, 0.0011, and 0.0012 were obtained for the training, testing, validation, and overall datasets, respectively. These values confirm that the discrepancies between predicted and experimental observations are extremely small, reflecting the excellent numerical precision achieved by the optimized model throughout the investigated experimental domain.

Equally important is the consistency of these statistical indicators among the different datasets. The negligible variation observed in both *R* and RMSE demonstrates that the predictive accuracy was maintained when the model was applied to unseen data, indicating that the optimized DT_LSBoost model generalized well beyond the calibration dataset. This behavior suggests that the optimization strategy successfully controlled model complexity while preserving high predictive capability, thereby minimizing the likelihood of significant overfitting.

**Fig. 18:**
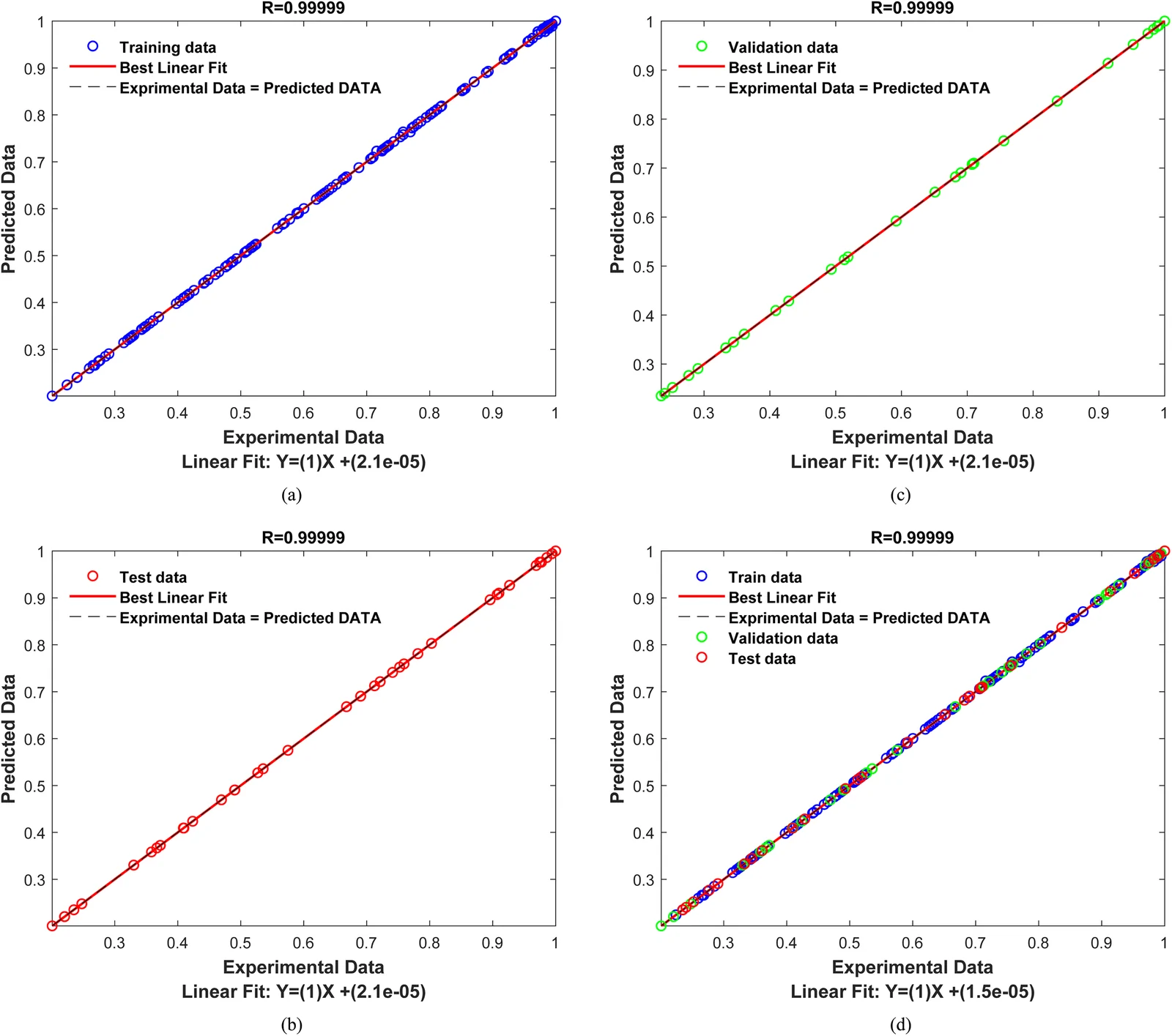
Correlation between experimental results and DT_LSBOOST model predictions: (a) training set, (b) test set, (c) validation set, and (d) combined data.

A graphical assessment of model performance is presented in Fig. 18, where the experimental values are compared with the corresponding DT_LSBoost predictions for the training, testing, validation, and combined datasets. In all cases, the data points are closely distributed around the line of perfect agreement, with no noticeable systematic deviation or increasing error dispersion. The uniform distribution of the residuals over the entire prediction range further confirms the stability and robustness of the developed model. Therefore, both the statistical indicators and the graphical analysis demonstrate that the optimized DT_LSBoost model provides accurate and reliable predictions of diclofenac photocatalytic degradation under the investigated operating conditions.

#### 3.7.1. Residues study

The predictive quality of a machine-learning model cannot be evaluated solely through global statistical metrics such as the correlation coefficient or RMSE. A complementary examination of the residuals is essential because it provides detailed information regarding the distribution of prediction errors, the existence of systematic deviations, and the overall stability of the developed model.^86–89^ In this work, the residuals were calculated as the difference between the experimentally measured responses and the corresponding DT_LSBoost predictions. Their analysis provides an additional validation of the model by revealing whether the prediction errors occur randomly or follow identifiable patterns that may indicate model inadequacies.^90,91^

**Fig. 19:**
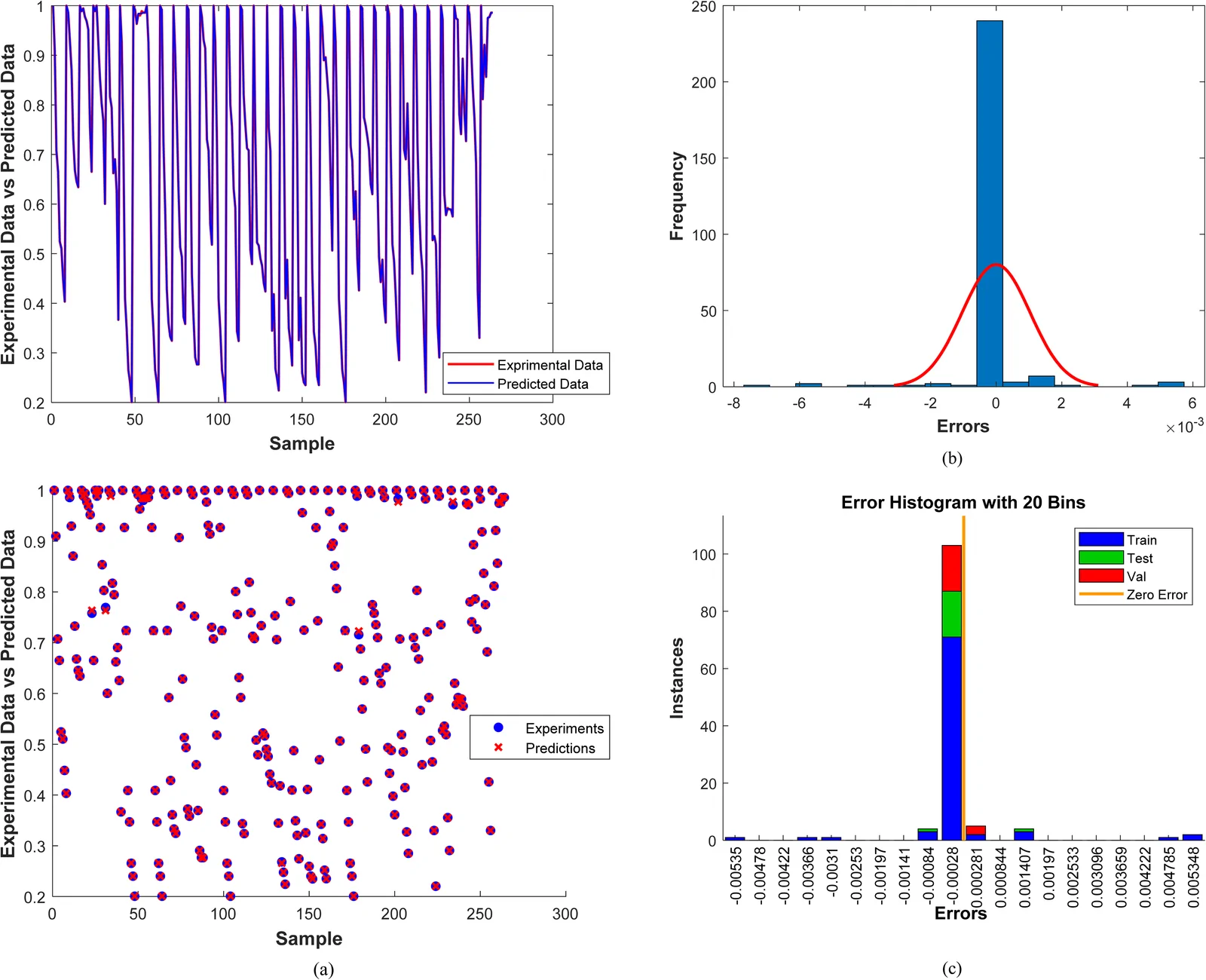
Residual diagnostics of the optimized DT_LSBoost model: (a) parity plot between experimental and predicted values, (b) frequency distribution of prediction residuals, and (c) residual distribution for the training, testing, and validation datasets.

Fig. 19(a) Presents the parity plot comparing the experimental observations with the corresponding predicted values. The data points are concentrated along the diagonal line representing perfect agreement, indicating that the model accurately reproduces the experimental behavior over the entire response range. The absence of noticeable curvature or directional deviation from the identity line suggests that the developed model does not exhibit systematic prediction bias and maintains consistent accuracy for both low and high response values. Additional insight into model performance is obtained from the residual histogram shown in Fig. 19(b). The distribution is sharply centered around zero, demonstrating that the prediction errors remain extremely small for most experimental observations. Among the 264 analyzed samples, 247 residuals fall within the narrow interval extending from −8 × 10^−3^ to 6 × 10^−3^, indicating that only minor discrepancies exist between experimental and estimated values. Furthermore, the nearly symmetric shape of the histogram reveals a balanced distribution of positive and negative residuals, confirming that the model does not consistently overestimate or underestimate the target variable. The evolution of the residuals throughout the model development procedure was further investigated by separately analyzing the training, testing, and validation datasets, as illustrated in Fig. 19(c). For each subset, the residuals are randomly dispersed around the zero-reference line with no visible trend, progressive increase in variance, or clustering of errors. Such behavior demonstrates that the prediction accuracy remains essentially unchanged regardless of the dataset considered. The comparable residual distributions observed for the three subsets indicate that the optimized DT_LSBoost model preserves its predictive capability when applied to unseen observations, highlighting its strong generalization performance and providing no evidence of significant overfitting. Overall, the residual diagnostics consistently support the statistical results obtained previously. The narrow dispersion of prediction errors, their random distribution around zero, and the absence of systematic error patterns collectively demonstrate that the optimized DT_LSBoost model provides stable, unbiased, and highly accurate predictions throughout the investigated experimental domain. These findings confirm that the developed model is suitable for reliable prediction of diclofenac photocatalytic degradation under different operating conditions.

#### 3.7.2. Tool for predictive modeling

**Fig. 20:**
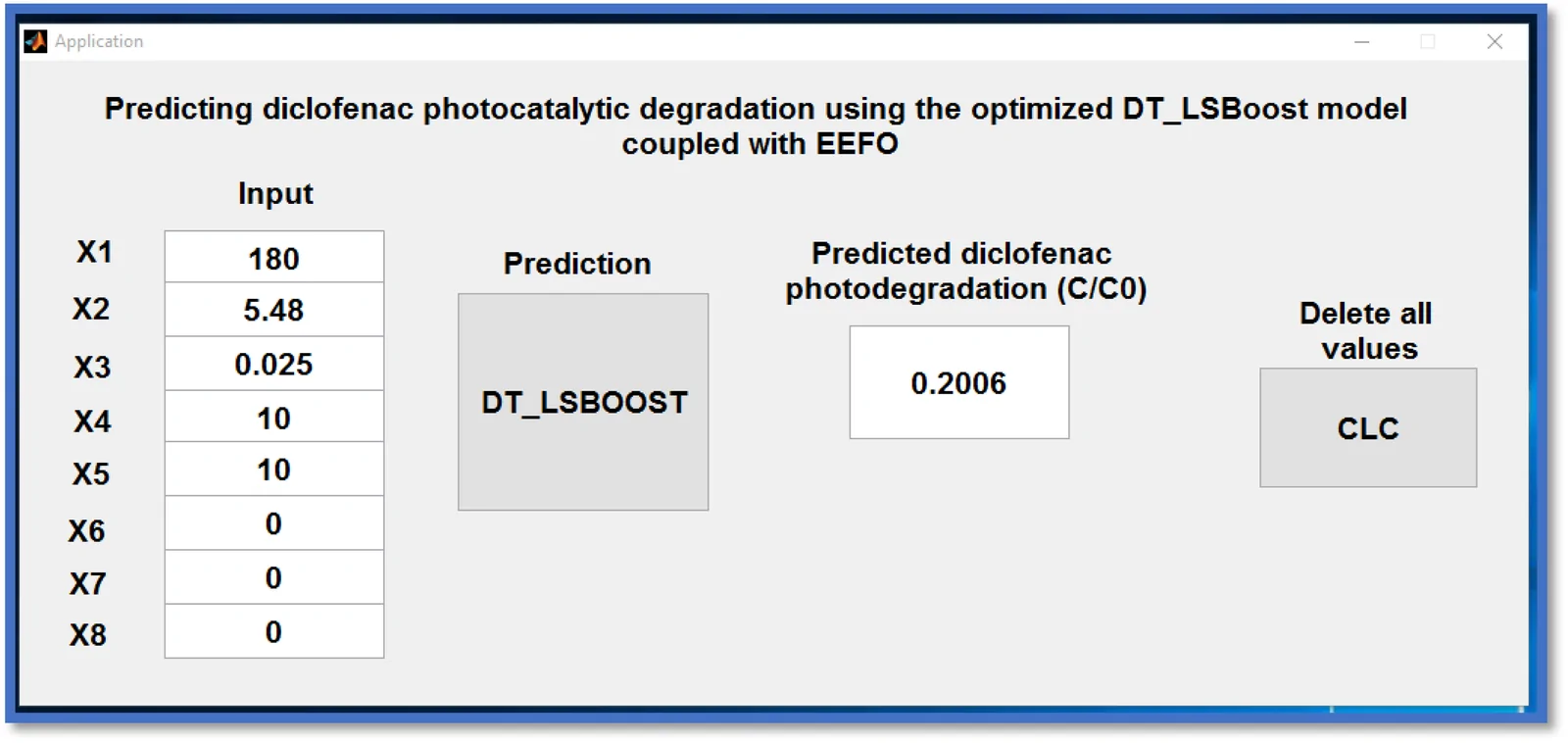
Development of a MATLAB-based interface for photodegradation prediction of diclofenac using DT_LSBOOST coupled with EEFO optimization.

To enhance the applicability of the proposed model beyond the research environment, a graphical user interface (GUI) was implemented using MATLAB Fig. 20.The developed application integrates the optimized DT_LSBoost model into an intuitive platform that allows users to specify the experimental operating conditions and instantly obtain predictions of the residual diclofenac concentration (*C*/*C*_0_) and degradation efficiency. By eliminating the need to directly interact with the machine learning code, the GUI significantly simplifies model utilization and enables rapid evaluation of different operational scenarios. Consequently, the application represents a practical decision support system that can assist researchers and engineers in process optimization, experimental planning, and photocatalytic performance assessment.

### 3.8. Practical implications and real wastewater treatment

The photocatalytic process using Ag/MgO NCs for treating wastewater from pharmaceutical facilities shows significant potential in mineralizing complex organic contaminants and pathogens. Key considerations for scaling this process include:

(1) Pilot-scale optimization: conducting pilot studies is essential to refine the Ag/MgO system under actual operational conditions. This involves optimizing variables such as catalyst dosage, solar light intensity (natural or artificial UV/Visible sources), and residence time, which can vary greatly with different wastewater compositions.

(2) Wastewater characterization: in-depth characterization of wastewater is crucial to identify pharmaceutical pollutants and organic loads, facilitating an understanding of how competing substances may adsorb on Ag/MgO and ensuring compliance with strict discharge regulations for hospital and industrial effluents.

(3) Economic viability: a comprehensive cost-benefit analysis is necessary to evaluate this photocatalytic approach against traditional treatment methods. Benefits include the low-cost green synthesis of Ag/MgO, the potential for catalyst recycling, and utilizing natural sunlight as an economical energy source.

(4) Environmental impact and sustainability: an environmental impact assessment (EIA) is required to ensure the process aligns with sustainability goals.^92^ Using non-toxic, green-synthesized nanoparticles mitigates the risk of secondary environmental issues, promoting a “green” chemistry philosophy in water treatment.

## 4. Reusability and stability of 10% Ag/MgO NCs

Recyclability and stability are critical factors for the long-term, cost-effective application of heterogeneous photocatalysts in continuous water remediation. To evaluate the operational durability of the synthesized 10% Ag/MgO NCs, its photocatalytic degradation performance toward diclofenac (DCF) was assessed over consecutive five runs under natural sunlight irradiation.

**Fig. 21:**
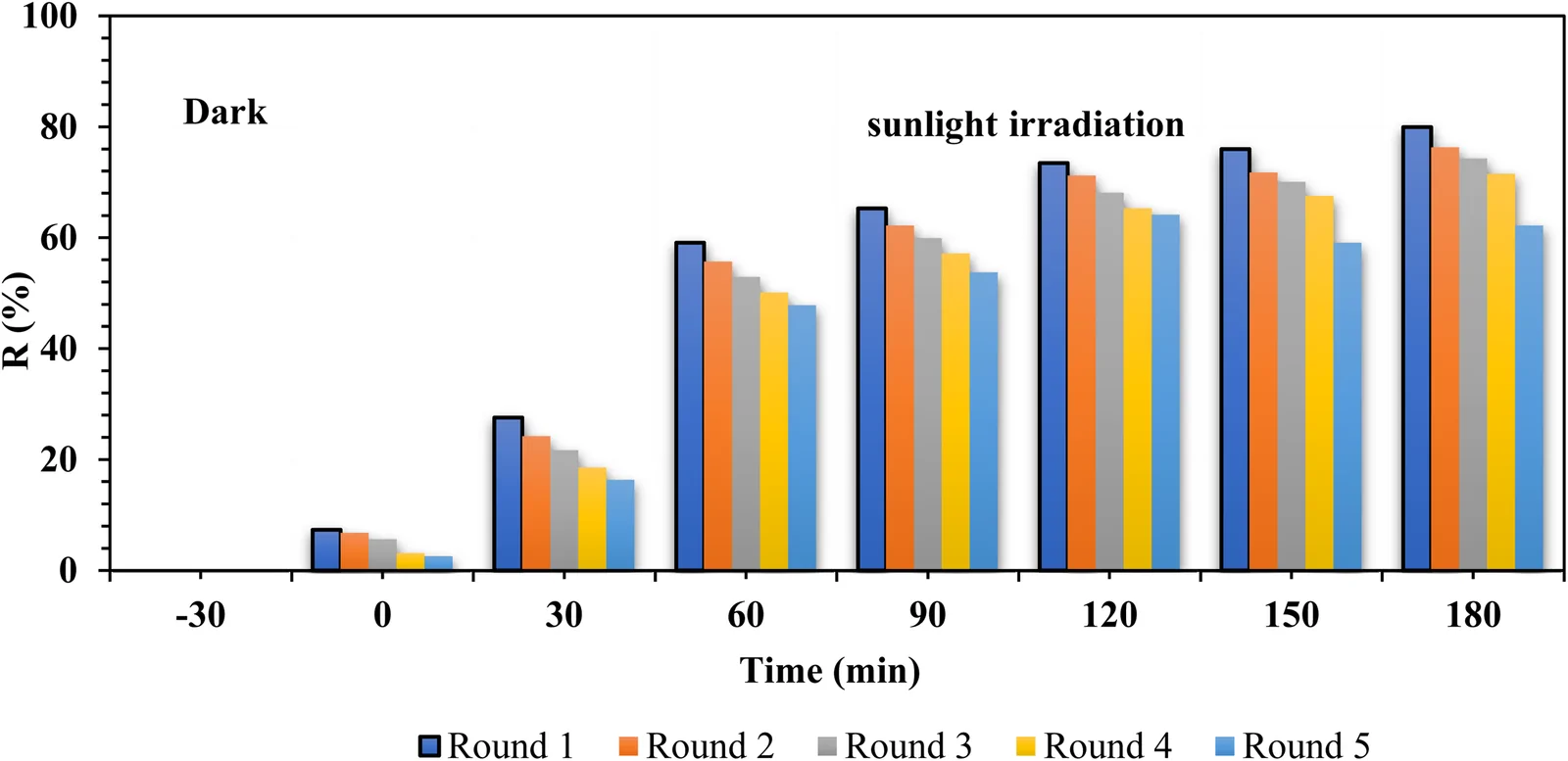
Reusability curve of DCF photocatalysis under direct sunlight onto 10%Ag/MgO NCs.

As illustrated in Fig. 21, the 10% Ag/MgO NCs demonstrated robust stability across successive cycles while maintaining high catalytic efficiency. The slight decrease in photodegradation efficiency over multiple uses can be attributed to minor catalyst mass loss during recovery steps and the progressive accumulation of recalcitrant degradation intermediates occupying active surface sites. Nevertheless, the nanocomposite preserved substantial photocatalytic activity without noticeable structural deactivation, highlighting its strong reusability and potential for scalable solar-driven pharmaceutical wastewater treatment.

## 5. Conclusion

A sustainable and integrated strategy combining green nanotechnology, solar-driven photocatalysis, and explainable machine learning was successfully developed for the removal of diclofenac (DCF) from water. Ag/MgO nanocomposites were green synthesized using *Capsicum annuum* L. seed extract as a natural reducing and capping agent, providing an environmentally benign route for the preparation of the photocatalytic materials. Physicochemical characterization confirmed the successful formation of crystalline Ag/MgO nanocomposites, with Ag incorporation decreasing the crystallite size from 12.0 to 9.0 nm and extending the optical absorption toward the visible region, with an apparent threshold of 1.83 eV. These modifications enhanced the ability of the material to utilize solar irradiation.

The photocatalytic experiments demonstrated that Ag incorporation significantly improved the degradation of DCF compared with pristine MgO. Among the investigated materials, 10% Ag/MgO exhibited the highest photocatalytic activity, achieving 80% degradation of 10 mg L^−1^ DCF within 180 min under natural sunlight under the optimized conditions. The systematic investigation of irradiation time, catalyst dosage, Ag loading, solution pH, initial DCF concentration, electrolyte type and concentration, and irradiation source demonstrated the important influence of operating conditions on photocatalytic performance. Radical trapping experiments identified hydroxyl radicals (˙OH) as the predominant reactive species, while metallic Ag acted as an electron sink, suppressing electron–hole recombination and facilitating the generation of reactive oxygen species. These results highlight the beneficial role of Ag incorporation in improving both the optical response and charge-transfer behavior of MgO.

In addition to the experimental investigation, an EEFO-optimized DT-LSBoost model was developed to predict DCF degradation efficiency from the investigated operating parameters. The optimized model exhibited excellent predictive performance, with *R* = 0.9999 and RMSE = 1.2 × 10^−3^, while residual analysis supported its robustness and generalization capability. The development of a MATLAB-based graphical user interface further transformed the predictive model into a practical decision-support tool, enabling rapid estimation of photocatalytic performance under different operating conditions without requiring direct interaction with the underlying machine-learning algorithm.

Overall, this study demonstrates that integrating green-synthesized Ag/MgO nanocomposites, solar photocatalysis, and explainable machine learning provides an effective and sustainable framework for diclofenac removal and photocatalytic process optimization. The proposed approach combines experimental treatment with data-driven prediction, offering the potential to reduce experimental effort while supporting the design and optimization of solar-driven water-treatment processes. Future studies should focus on catalyst stability and reusability, identification of degradation pathways and transformation products, ecotoxicological assessment, validation in real wastewater matrices, and extension of the predictive framework to other photocatalytic systems and emerging pharmaceutical contaminants.

## Conflicts of interest

There are no conflicts to declare.

## Data Availability

All data supporting the findings of this study are included within the article.
